# I/Pu reveals Earth mainly accreted from volatile-poor differentiated planetesimals

**DOI:** 10.1126/sciadv.adg9213

**Published:** 2023-07-05

**Authors:** Weiyi Liu, Yigang Zhang, François. L. H. Tissot, Guillaume Avice, Zhilin Ye, Qing-Zhu Yin

**Affiliations:** ^1^ The Isotoparium, Division of Geological and Planetary Sciences, California Institute of Technology, Pasadena, CA 91125, USA.; ^2^ Key Laboratory of Computational Geodynamics, College of Earth and Planetary Sciences, University of Chinese Academy of Sciences, Beijing 100049, China.; ^3^ Université Paris Cité, Institut de physique du globe de Paris, CNRS, Paris F-75005, France.; ^4^ Key Laboratory of High-Temperature and High-Pressure Study of the Earth’s Interior, Institute of Geochemistry, Chinese Academy of Sciences, Guiyang, Guizhou 550081, China.; ^5^ Department of Earth and Planetary Sciences, University of California, Davis, CA 95616, USA.

## Abstract

The observation that mid-ocean ridge basalts had ~3× higher iodine/plutonium ratios (inferred from xenon isotopes) compared to ocean island basalts holds critical insights into Earth’s accretion. Understanding whether this difference stems from core formation alone or heterogeneous accretion is, however, hindered by the unknown geochemical behavior of plutonium during core formation. Here, we use first-principles molecular dynamics to quantify the metal-silicate partition coefficients of iodine and plutonium during core formation and find that both iodine and plutonium partly partition into metal liquid. Using multistage core formation modeling, we show that core formation alone is unlikely to explain the iodine/plutonium difference between mantle reservoirs. Instead, our results reveal a heterogeneous accretion history, whereby predominant accretion of volatile-poor differentiated planetesimals was followed by a secondary phase of accretion of volatile-rich undifferentiated meteorites. This implies that Earth inherited part of its volatiles, including its water, from late accretion of chondrites, with a notable carbonaceous chondrite contribution.

## Teaser

The co-partitioning of I and Pu during metal-silicate differentiation reveals a heterogeneous accretion history of the Earth.

## INTRODUCTION

Earth must have accreted from diverse materials, but the nature and temporal sequence of delivery of these potential building blocks remain heavily debated (*1*–*6*). To investigate these questions, the isotopes of xenon (Xe), the heaviest stable noble gas, are particularly useful. Because ^129^Xe* comes from radioactive beta decay of now extinct volatile ^129^I (*t*_1/2_ = 15.7 Ma) and ^136^Xe*_Pu_ comes from spontaneous fission of extinct refractory ^244^Pu (*t*_1/2_ = 80 Ma), the ^129^Xe*/^136^Xe*_Pu_ ratio evolves as a function of both time and reservoirs compositions (i.e., I/Pu ratio) early in Earth’s history. Hence, the study of the ^129^Xe*/^136^Xe*_Pu_ in silicate reservoirs of Earth has the potential to place strong constraints on Earth’s accretion and evolution (*7*–*13*). According to recent high-precision analyses of Xe isotopes, ocean island basalt (OIB) samples [plume mantle sources, originating from as deep as the core mantle boundary (CMB)] display a uniformly low ^129^Xe*/^136^Xe*_Pu_ (by a factor of ~2.8) compared to mid-ocean ridge basalt (MORB) samples (upper mantle sources) (*7*–*9*, *13*). Previous work has shown that these signatures cannot simply result from shallow atmospheric contamination, mixing between subducted atmospheric Xe and MORB Xe, and/or different closure ages of Xe loss between the shallow and deep mantle reservoirs (*7*, *9*, *13*). Instead, the low ^129^Xe*/^136^Xe*_Pu_ in the plume reservoir indicates that a low I/Pu was established before ^129^I extinction (i.e., first ~80 to 100 Ma of the Solar System) and has been preserved thereafter, avoiding rehomogenization, for about 4.45 billion years (*7*, *10*, *13*).

Because iodine is thought to be retained since the earliest stages of accretion (*14*, *15*), two main competing mechanisms have been proposed to explain the I/Pu contrast between MORB and OIB mantle reservoirs: (i) a heterogeneous volatile accretion history for Earth (*7*, *10*) or (ii) a homogeneous volatile accretion history where partitioning of iodine into liquid metal during core formation was taken into account (*11*, *16*). Both models have important ramifications for our understanding of Earth’s evolution. In the first case, volatile elements (including iodine) would be depleted in early accreted materials compared to later building blocks of Earth and inefficiently mixed into Earth’s whole mantle. In the second scenario, a change in the nature of Earth’s building blocks is not required because the iodine depletion of the deeper mantle could be achieved through episodes of high-pressure core formation. However, both models also suffer from notable shortcomings. The heterogeneous accretion models (*7*, *10*) did not consider the impact of core formation processes, and neither model considered the geochemical behavior of plutonium at the high *P*-*T* (pressure-temperature) conditions relevant to core formation. This last parameter could exert a strong control on the evolution of I/Pu ratios within Earth’s reservoirs during its accretion. In the near absence of experimental data on metal-silicate Pu partitioning (*17*)—due to its highly radioactive nature and lack of access to suitable amount to perform these experiments—Pu has been implicitly assumed to be a rock-loving (lithophile) element independent of temperature and pressure throughout the planetary accretion and core formation processes.

To remedy this situation and assess whether core formation alone without a change in volatile content of building blocks could explain the difference in I/Pu ratios between MORBs and OIBs, we used the two-phase first-principles molecular dynamics (FPMD) method (*18*–*20*) to determine the partition coefficient of I and Pu between liquid iron and silicate melt. This method has successfully predicted the partition coefficients of C, He, Mg, Si, and O (*18*, *20*). Our newly derived partition coefficients can then be used in multistage core formation model to assess whether I/Pu could be efficiently fractionated under different accretion scenarios. Enhanced by a thorough compilation and analysis of available meteorite xenon isotope data, we present a model for the accretion history of Earth based on I/Pu evidence.

## RESULTS

The FPMD simulations were ran at pressures ranging from 25 to 85 GPa along the mantle liquidus (*21*). These values were chosen to cover the range of *P*-*T* conditions relevant to Earth’s accretion according to the current prevailing literature (*22*, *23*). Each simulation started with 256 Mg, Si, O, and Fe atoms (in proportions reflecting the bulk Earth composition, see Materials and Methods) and 4 I or Pu atoms placed randomly in the simulation boxes. After 30,000 steps of equilibration, the atoms are segregated into two phases, a liquid iron phase and a silicate melt phase. A polyhedron “alpha shape” of Fe cluster is constructed to mark the boundary between the two phases (fig. S1). Atomic configurations from the next 30,000 to 50,000 simulation steps are used to calculate the average compositions of the liquid iron and silicate melt phases (tables S1 and S2).

Partitioning of plutonium and iodine between silicate melt and metal liquid takes place according to the following reactions (see Materials and Methods)

$$I^{\mathrm{silicate}}⇋I^{\mathrm{metal}}$$ (1)

$${\mathrm{Pu}O}_{\frac{3}{2}}^{\mathrm{silicate}}⇋{\mathrm{Pu}}^{\mathrm{metal}}+\frac{3}{2}O^{\mathrm{metal}}$$ (2)

The corresponding equilibrium constants, *K*_Pu_ and *K*_I_, are of the form, ln(*K*) = *a* + *b*/*T* + *cP*/*T*, where *T* is temperature (in Kelvin) and *P* is pressure (in gigapascal), and were derived by fitting the exchange coefficients (*K*_D_) of our data and previous laser-heated diamond anvil cell (LH-DAC) experimental data of iodine (*11*) using least-squares regression, yielding

$$\ln(K_{I})=(1.34\pm 1.51)-(13.4\pm 4.8)\frac{1000}{T}+(131\pm 48)\frac{P}{T}$$ (3)

$$\ln(K_{\mathrm{Pu}})=(11.1\pm 1.1)-(56.8\pm 3.5)\frac{1000}{T}$$ (4)

**Fig. 1.**
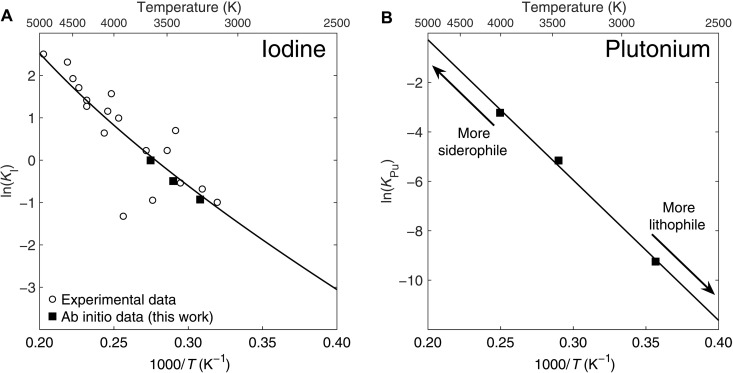
High-pressure metal-silicate equilibrium constants for iodine and plutonium, as a function of temperature. Filled squares are ab initio data from this study (see tables S1 and S2), while open circles denote experimental data from (*11*). The curves are least-squares fits to all available data using Eqs. 3 and 4. (**A**) Equilibrium constant of iodine. All data are corrected to the cold mantle liquidus geotherm constructed through the melting of chondritic mantle (*21*). (**B**) Equilibrium constant of plutonium. The *P*-*T* conditions of the FPMD simulations were chosen to be most relevant to the accretion model and avoid extrapolation.

The first-principles calculation data that we obtained for iodine are fully consistent with previous experimental data (Fig. 1A), further justifying the adequacy of our FPMD method for estimating elemental partition coefficients (*18*, *20*). Our results show that, similar to iodine, Pu becomes less lithophile at a higher temperature (Fig. 1B). This temperature dependency of *K*_Pu_ is in line with the similar behavior of another actinide, uranium, observed in LH-DAC experiments (*24*, *25*).

## DISCUSSION

Both I and Pu tend to partition into the metal phase under high pressures and temperatures relevant to core formation (Fig. 1). However, given that the I and Pu partition coefficients (*D = X*^metal^*/X*^silicate^) have different *P*-*T*-$X_{O}^{\mathrm{metal}}$ (oxygen molal fraction in metal phase) dependency (figs. S2 and S3), the silicate mantle would lose I and Pu to the core in variable proportions during metal-silicate equilibration events. To assess whether core formation could sufficiently fractionate I and Pu to explain the ^129^Xe*/^136^Xe*_Pu_ offsets between the OIB and the MORB mantle sources, we run a series of multistage core formation simulations (*22*) (see Materials and Methods). To explain the lower ^129^Xe*/^136^Xe*_Pu_ in the plume mantle, a factor of 2.8 depletion of I/Pu would be required, assuming a synchronous closure time of Xe loss for the whole mantle (*9*, *11*). This is a conservative assumption because (i) prolonged Xe loss from the mantle to the atmosphere would tend to decrease the upper mantle ^129^Xe*/^136^Xe*_Pu_ and require an even higher initial I/Pu ratio in the upper mantle to account for the observed contrast between MORB and OIB ^129^Xe*/^136^Xe*_Pu_ ratios (*13*) and (ii) a later closure time of the plume mantle source compared to the upper mantle would be physically difficult to explain.

### Homogeneous volatile accretion

**Fig. 2.**
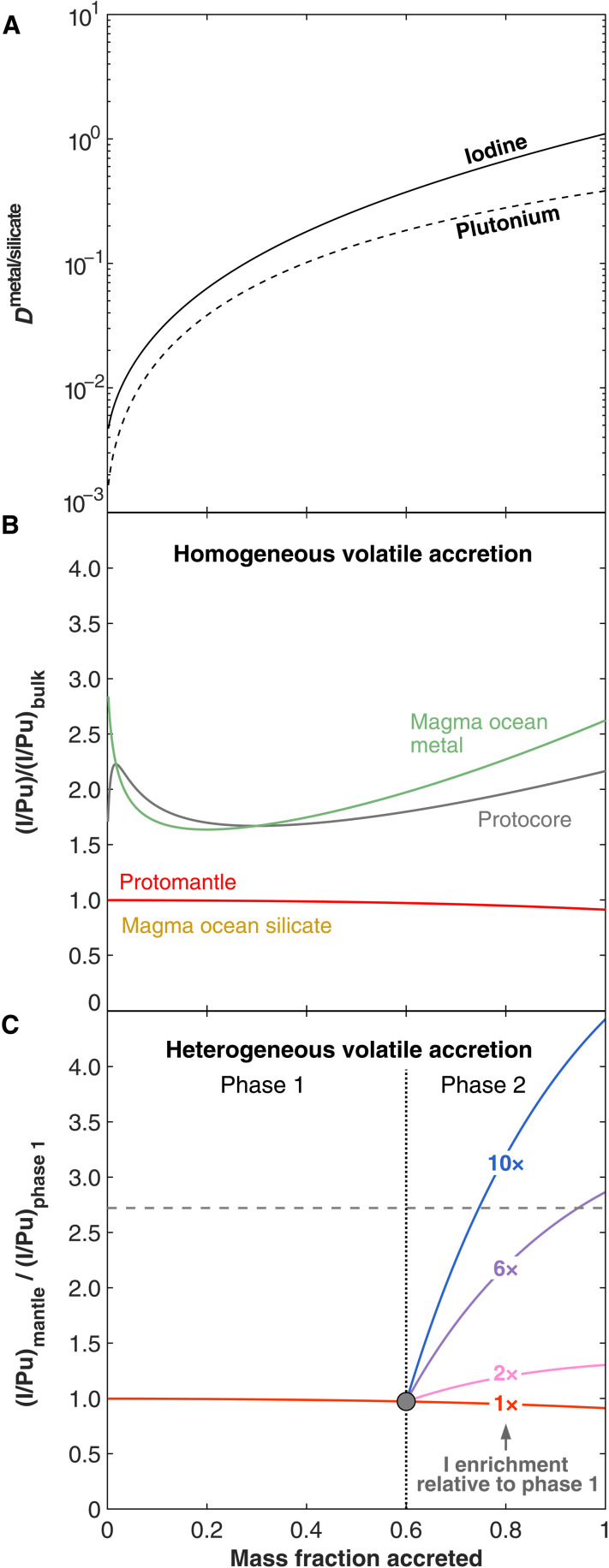
Changes in I and Pu partitioning behaviors and their impact on the I/Pu of Earth’s reservoirs as a function of the mass accreted to Earth. (**A**) Partition coefficients (*D = X*^metal^*/X*^silicate^) of iodine and plutonium between silicate melt and metal liquid during core formation processes. The simulation used the fiducial multistage model (i.e., cold liquidus geotherm, 60-GPa maximum pressure, and reduced conditions; see Materials and Methods for details). On the basis of these partition coefficients, change in the I/Pu ratio in terrestrial reservoirs was calculated assuming (**B**) a homogeneous and (**C**) a heterogeneous volatile accretion scenario. In (B), all materials accreted to Earth have identical I/Pu ratio, and core formation results in nearly invariant mantellic (mantle and silicate melt in magma oceans) I/Pu ratios during accretion. The high I/Pu in metal fractions (core and liquid metal in magma oceans) rules out a core/metal contribution to the plume mantle as the source of the low ^129^Xe*/^136^Xe*_Pu_. The I/Pu ratios (*y* axis) are normalized to the bulk I/Pu ratio of the building block materials. In (C), materials in the later stages of accretion (phase 2) are enriched in iodine compared to those in the earlier stages of accretion (phase 1; see enrichment factor on each colored curve). The *y* axis shows the mantle I/Pu ratio normalized to the bulk I/Pu ratio of the material accreted during phase 1. Accretion of volatile-enriched materials in phase 2 can result in an increase in I/Pu ratio in the final mantle relative to the mantle at the end of phase 1 by a factor of 2.8 (horizontal dashed line) or more, which is the lowermost value needed to explain the ^129^Xe*/^136^Xe*_Pu_ offsets between the MORBs and OIBs.

First, we assume a homogeneous volatile accretion scenario, meaning that in each simulation, all building blocks are assumed to have the same I/Pu ratio (i.e., volatile content). Equilibrium pressure, temperature, and oxygen fugacity (*f*o_2_) during accretion were varied to test the sensitivity of the simulation outcomes (see Materials and Methods and figs. S4 to S6). Under the assumption of homogeneous volatile accretion (i.e., constant I/Pu in Earth’s building blocks), we find that core formation processes do not result in substantial fractionation of I from Pu in Earth’s mantle. I and Pu partition into the metal phase very similarly during core formation processes (e.g., Fig. 2A and fig. S7), and mantle I/Pu ratios barely vary during accretion, with the mantle I/Pu ratio of the fully accreted Earth being only 8% lower than that of the initial protomantle (Fig. 2B). This is the case even when the equilibrium pressure of last accretion is assumed to be the upper bound (80 GPa) needed to generate a distribution of other moderately siderophile elements (e.g., Ni, V, Co, and Cr) consistent with observations (*22*) (fig. S4). Even assuming an extreme accretion scenario with higher equilibration pressure in the middle stage of accretion compared to the late stages (*11*), the maximum difference between the accreting and the final mantle I/Pu ratios is only ~25% (fig. S4, B and C). Hence, the isotopic Xe signature in the plume mantle source is unlikely to be readily explained by episodes of a high equilibrium pressure during any stage of a homogeneous accretion.

The above calculations were run assuming accretion starting from reduced building materials, but changes in *f*o_2_ during accretion [simulated here using prescribed evolution curves for the FeO content of the magma ocean (*22*)] could have a notable effect on the I/Pu ratio of the mantle of proto-Earth (fig. S5). This effect of changes in *f*o_2_ depends on the assumed *T* of metal-silicate equilibration. When equilibration occurs along the mantle liquidus geotherm based on the melting of a chondritic mantle (cold liquidus geotherm hereafter) (*21*), the I/Pu ratio for Earth’s mantle monotonically decreases throughout Earth’s accretion (fig. S5A). Regardless of the *f*o_2_ conditions, under the cold liquidus geotherm, isolation of an early formed mantle reservoir would yield higher I/Pu ratio in OIBs than MORBs, the opposite of what is observed. In contrast, at the very high temperatures following the liquidus geotherm constructed through the melting profiles of peridotite (hot liquidus geotherm hereafter) (*26*) and for the most oxidized evolution scenarios [log(*f*o_2_) ~ IW-0.6, where IW is the iron-wüstite buffer], metal-silicate equilibration would lead to a 4.5 times lower I/Pu ratio in the middle stages of accretion compared to the final mantle (fig. S5B). If some part of the mantle in this intermediate stage was not involved in the subsequent accretion process and preserved throughout Earth’s history, then these relics of proto-Earth’s mantle could explain the lower ^129^Xe*/^136^Xe*_Pu_ in the plume mantle. However, elemental and isotopic evidence suggests that Earth mainly accreted under less oxidizing conditions (i.e., between IW-4.5 and IW-1.5) (*1*, *2*, *22*, *23*, *27*, *28*), not to mention that accretion under oxidizing conditions would yield a much smaller terrestrial core than observed (*29*). Considering only the plausible range of *f*o_2_ relevant to Earth’s accretion (starting from a value between IW-4.5 and IW-1.5), even in the hot liquidus geotherm case, our simulations reveal that iodine is unlikely to be sufficiently fractionated from Pu during a homogeneous volatile accretion to explain the 2.8 times lower I/Pu recorded in the plume mantle (fig. S5B).

Incorporation of intrusions from Earth’s liquid outer core and/or suspended liquid metallic droplets into part of the silicate mantle has been proposed as the possible source of the plume mantle (*30*). If metal-silicate equilibration happened at temperatures following the cold liquidus geotherm, then both Earth’s core and the liquid metal in the magma ocean would have consistently higher I/Pu ratios than the values in the bulk mantle (Fig. 2B). In this scenario, any core/metal contribution to the plume mantle would lead to a higher ^129^Xe*/^136^Xe*_Pu_ after decay of ^129^I and ^244^Pu, again the opposite of what is observed between MORBs and OIBs. Accretion under the hot liquidus geotherm, treated as the upper limit for the equilibrium temperatures, can produce I/Pu ratio two times lower in the magma ocean liquid metal (i.e., the metal after silicate-metal equilibration but before segregation into Earth’s core) or in the protocore compared to the mantle during the middle and late stages of accretion (fig. S6). This value is only 30% lower than the factor of 2.8 difference observed between OIBs and MORBs but assumes that the plume mantle Xe derives entirely from the core/metal. In the much more likely scenario where the plume mantle initially contained some Xe, incorporation of Xe from the metal/core could not produce the 2.8 times offset observed between the MORB and plume mantle reservoirs. Overall, these results suggest that the Xe anomalies in the plume mantle are unlikely to be due to incorporation of Xe from the core and/or the presence of suspended droplets of magma ocean metal liquids that never settled to Earth’s core (*30*, *31*).

### Heterogeneous volatile accretion

We now consider a scenario of heterogeneous volatile accretion with core formation processes, whereby the volatile content (i.e., I/Pu ratio) of Earth’s building blocks is no longer kept constant over time. Moderately volatile siderophile elements in Earth’s mantle suggest that volatile elements may be delivered to Earth in the later stages of the accretion process (*2*, *32*–*34*), implying that the material accreted to Earth during early stages of accretion (phase 1) likely had a lower I/Pu ratio than material accreted in the later stages of accretion (phase 2) (see Fig. 2C). Specifically, our phase 2 includes not only the later stage of core formation process but also the late veneer (i.e., the last ~0.5% mass of accretion that dominates the highly siderophile element budget of bulk silicate Earth), as they are thought to have delivered the same volatile-rich (high I/Pu ratio) building block materials to Earth (*1*). Note that the late veneer does not participate in the core formation process, and the additional volatiles it delivers are directly added to the mantle. In these heterogeneous accretion scenarios, the I/Pu ratio in Earth’s mantle at the end of its accretion history (end of phase 2) could be much higher than the I/Pu ratio in the proto-Earth’s mantle at the end of phase 1. At the CMB, a small portion of the proto-Earth’s mantle before addition of volatile-rich material, if isolated from the subsequent accretion process and Earth’s dynamical evolution (*30*, *35*, *36*) thereafter, could be the source of the depleted I/Pu mantle reservoirs sampled by OIBs (Fig. 2C), while the rest of the mantle enriched by the subsequent phase 2 accretion processes would represent the reservoirs with higher I/Pu ratio sampled by MORBs.

**Fig. 3.**
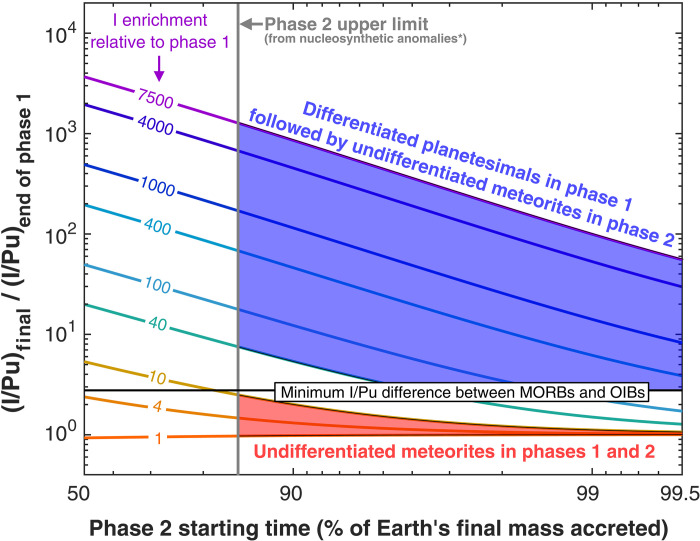
Final MORB mantle’s I/Pu ratio (normalized to the mantle I/Pu at the end of phase 1) as a function of the starting time of phase 2 of accretion. Each curve represents a different extent of iodine enrichment (curve labels) in the phase 2 materials relative to phase 1 materials. As phase 2 includes late veneer (last 0.5 wt % of accretion process), its starting time should be earlier than 99.5 wt % of Earth’s final mass accreted. Simulation conditions as in Fig. 2. The horizontal black line denotes a I/Pu ratio in the final mantle that is 2.8× higher than at the end of phase 1. The vertical line shows the upper limit (15 wt %) on the mass fraction of volatile-rich material accreted by Earth late in its history, as constrained by nucleosynthetic anomalies data (εMo and correlations with εCr, εNd, and εZr) on meteorites (*3*). Large I/Pu enrichments in the MORB mantle (compared to the mantle at the end of phase 1) are only achieved if volatile-poor, differentiated planetesimals (with I/Pu ratios of 40 to 7500× lower than chondrites; tables S4 and S5) constitute the main building blocks of Earth during phase 1 (blue area). In contrast, accretion histories involving undifferentiated chondrites (whose I/Pu ratios only differ by a factor of ~10; tables S4 and S5) as the main material in phase 1 (red area) fail to yield a 2.8× difference in mantellic I/Pu ratio. To date, there are no known planetary materials with I/Pu enrichment between 12 and 40× (tables S4 and S5) although these materials could produce large I/Pu enrichments in the MORB mantle.

Figure 2C shows an illustrative calculation of a heterogeneous volatile accretion history where addition of volatile-rich materials (with high I/Pu) starts after 60% of Earth’s accretion. In this example, the material accreted in phase 2 only needs to have ~6 times higher I/Pu than that accreted in phase 1 to explain the ^129^Xe*/^136^Xe*_Pu_ contrast between OIBs and MORBs. Evidently, onset of phase 2 at a later time would require a higher I/Pu in the late accreted material to still explain the 2.8 times difference in Xe isotope ratios between MORBs and OIBs. The relationship between the timing of onset of phase 2 (no later than 99.5 wt % of Earth’s final mass accreted as it includes late veneer) and the mantle’s I/Pu enrichment (i) after core formation processes (which could be treated as the endmember scenario in which the late veneer happened after ^129^I extinction and cannot deliver any ^129^Xe to Earth) and (ii) after the entire accretion history are respectively shown in fig. S8 and Fig. 3, along with independent constraints on these variables. Although some earlier works suggested that a large fraction (30 to 40 wt %) of Earth’s building blocks were volatile-rich, recent estimates (*3*) based on a multielement comparison between the isotopic composition of bulk Earth and meteorites indicate that volatile-rich materials account for <~15 wt % of the bulk Earth and most likely only ~4 wt %. On Fig. 3, the full range of allowable mass fraction of accretion of volatile-rich materials is thus shown to the right of the gray vertical line and reveals that I/Pu ratios in phase 2 materials must be more than an order of magnitude higher than in phase 1 materials to explain the minimum I/Pu differences between MORBs and OIBs (horizontal black line).

The high iodine enrichment, relative to Pu, in phase 2 materials indicates that accretion histories for Earth involving only chondrites are very unlikely. Deconvolving I-decay and Pu-fission Xe (see Materials and Methods) from thoroughly compiled available Xe isotopic data of different meteorites (tables S3) only suggests a small range (within a factor of ~10) of ^129^Xe*/^136^Xe*_Pu_ among chondrites (tables S4 and S5). Independently, the latest data in carbonaceous, enstatite, and ordinary chondrites show that iodine abundances do not vary substantially across the board (*37*), and the vast majority has iodine content within a factor of 10 of each other (*38*). Last, while some studies also suggested that comets could accrete to Earth during the late veneer (*39*, *40*), the fact that the iodine content of comets (67P/Churyumov-Gerasimenko) appears to be of same order of magnitude as that of chondrites (*37*, *38*, *40*) further strengthens the conclusion that chondrites are very unlikely to be the primary building block materials during phase 1.

### Accretion model of differentiated planetesimal

**Fig. 4.**
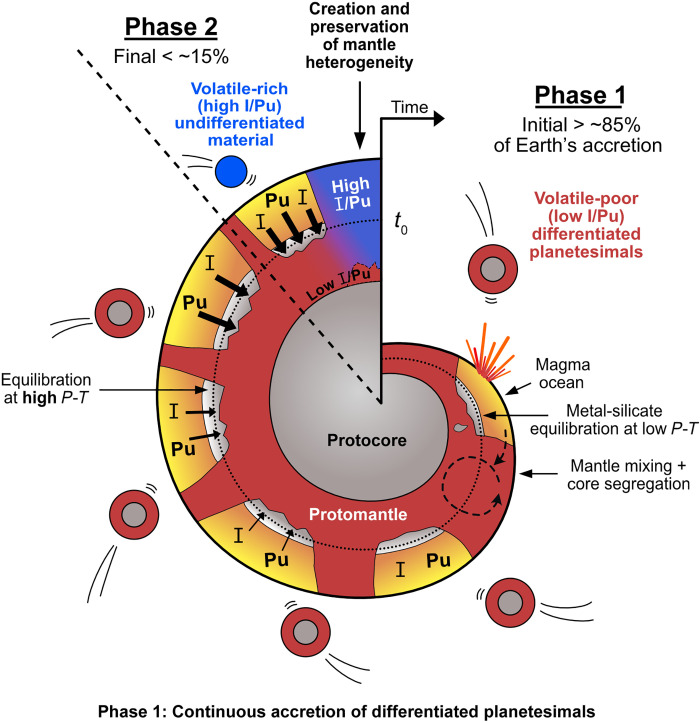
Schematic representation of the heterogeneous accretion history of Earth that is consistent with the more siderophile behavior of I and Pu at high *P*-*T* conditions (this work). As core formation alone does not result in I/Pu fractionations sufficient to explain the ~3 times lower ^129^Xe*/^136^Xe*_Pu_ ratio observed in OIBs compared to MORBs (Fig. 2B), a scenario of heterogeneous accretion has to be invoked in which volatile-depleted differentiated planetesimals constitute the main building blocks of Earth for most of its accretion history (phase 1), before addition of, comparatively, volatile-rich undifferentiated materials (chondrite and possibly comet) during the last stages of accretion (phase 2). Isolation and preservation, at the CMB, of a small portion of the proto-Earth’s mantle before addition of volatile-rich material would explain the lower I/Pu ratio of plume mantle, while the mantle involved in the last stages of the accretion would have higher, MORB-like, I/Pu ratios. Because the low I/Pu mantle would also have an inherently lower Mg/Si (fig. S9), its higher viscosity could help to be preserved at the CMB until today.

Compared to chondrites, achondrites have 40 to 7500 times lower ^129^Xe*/^136^Xe*_Pu_ (tables S4 and S5) and, thus, much lower I/Pu ratios. This high extent of iodine deficiency compared to plutonium in achondrites is in line with the idea that most parent bodies of achondrites are highly volatile-depleted and refractory-enriched (*5*). In a scenario where the parent bodies of achondrites represent Earth’s main building blocks in phase 1, the Xe isotopic offsets between MORBs and OIBs can be readily generated by late accretion of any type of chondrites. Most achondrites originated from volatile-depleted and refractory-enriched asteroids (*5*), which are themselves the remnants of differentiated planetesimals. Meanwhile, volatile-poor differentiated planetesimals have been suggested to be the archetypal first-generation planetary embryos formed in the innermost Solar System within <2 Ma of its formation (as defined by the age of calcium, aluminum-rich inclusions) (*41*). Our results thus support a heterogeneous accretion history of Earth, whereby early-formed, volatile-poor differentiated planetesimals represent Earth’s main building blocks (*6*) (>85%), and chondrites, which are comparatively volatile-rich, represent late-accreted materials and only account for a small fraction (<15%) of Earth’s mass (Fig. 4).

Isotopic constraints indicate that Earth accreted from both noncarbonaceous (volatile-poor inner Solar System material) and carbonaceous (volatile-rich outer Solar System material) materials (*3*, *42*). Our results further suggest that the noncarbonaceous material primarily consists of early differentiated planetesimals so as to create large enough volatile difference among Earth’s building blocks. Because silicon preferentially partitions into Earth’s core over magnesium at high pressure, the Mg/Si ratio in the mantle would increase during the accretion process (fig. S9). As a result, at the CMB, the portion of proto-Earth’s mantle isolated from the later stages of accretion would have a higher viscosity (due to lower Mg/Si ratio) compared to the final mantle (*43*): a feature that could help preserve the chemical records of early accretion of volatile-poor differentiated planetesimal at the base of the mantle until today. Our heterogeneous accretion model also readily explains the fact that present-day plume mantle reservoirs have a uniformly low ratio of potassium (a volatile, incompatible element) over uranium (a refractory, incompatible element) compared to the MORB mantle, without resorting to complex geochemical or geodynamical processes such as crust recycling or mantle convection (*44*, *45*). Last, our model sheds light on the origin of Earth’s water, as it requires that chondrites represent the main material delivered to Earth in the last 1 to 15% of its accretion (*3*, *6*). Independent constraints from Mo nucleosynthetic anomalies require these late accreted materials to come from the carbonaceous supergroup (*3*). Together, these results indicate that carbonaceous chondrites must have represented a non-negligible fraction of the volatile-enriched materials in phase 2 and, thus, play a substantial role in the water delivery to Earth. This is in agreement with recent evidence from H and N isotopes that independently point to delivery of 4 to 15% of CI-like materials to Earth during the late stages of accretion (*4*). A coherent picture is therefore emerging where Earth accreted mostly dry and gained its water and volatiles only late in its accretion history, highlighting the impact that small differences in formation history (e.g., changes in the nature of minor building blocks) can have on a planet’s evolution and habitability.

## MATERIALS AND METHODS

### FPMD simulations

Calculations were performed using the two-phase FPMD method following (*18*–*20*) and using the ab initio total energy and molecular dynamics program VASP (*46*). The projector-augmented wave potentials (*47*, *48*) were used together with the generalized gradient approximation of the exchange-correlation potential (*49*) in which 6 valence electrons are considered for O (2s^2^2p^4^), 2 for Mg (3s^2^), 14 for Fe (3p^6^3d^6^4s^2^), 4 for Si (3s^2^3p^2^), 7 for I (5s^2^5p^5^), and 16 for Pu (6s^2^6p^6^6d^2^5f^4^7s^2^). The plane-wave basis set cutoff was 400 eV. The accuracy for electronic self-consistent iteration was 10^−4^ eV. The Brillouin zone sampling was performed only at the gamma point, and the Fermi-Dirac smearing was used to consider the temperature effect. Molecular dynamics simulations were performed in the NVT canonical ensemble (i.e., number of atoms, volume, and temperature remain constant during simulation) with a time step of 1 fs. Because of the complex nature of the 5f electron states for Pu, the splitting of the orbital energy level under symmetrical crystal structures might cause spin crossover (*50*, *51*). However, it would be hard to form symmetrical configuration around Pu in silicate melt or iron metal liquid phases considering the size of Pu. Furthermore, the high pressure and temperature would favor Pu in the nonmagnetic low spin state even if the 5f could split in liquid (*51*). Therefore, in consideration of the time consumptions of our long-time (260 atoms and more than 60,000 steps) FPMD running, we did not perform spin-polarized calculations in this work.

The simulation box contained 260 atoms. Oxygen (O), Mg, Fe, and Si represent 256 of these atoms, and the other 4 atoms are I or Pu. The relative proportions of O, Mg, Fe, and Si were set to match bulk Earth compositional models from (*52*). Most simulations used the O-bearing Earth composition model, which contains 127 O, 51 Mg, 44 Fe, and 34 Si atoms, while the simulation run named “I3” (see table S1) used the Si-bearing Earth composition model, which has same number of Mg and Fe atoms but contains 123 O and 38 Si atoms. The size effect (i.e., number of atoms) has been tested in (*19*).

The atoms were randomly distributed in a simulation cell, and ~30,000 time steps were used to allow for segregation of liquid iron and silicate melt and for the system to reach equilibrium. Another 30,000 to 50,000 steps were used to calculate the average compositions of the liquid iron and silicate melt phases. For calculating composition of the Fe phase, a polyhedron alpha shape (a term used in computational geometry with smaller values of alpha describing more details of an object) was first constructed for the Fe cluster using the randomized incremental algorithm (*53*). The alpha shape is derived from the convex hull of iron cluster using Delaunay triangulation of all Fe atoms and describes the detail of the iron cluster surface by sifting the Delaunay tetrahedral sets according to the radius of their circumscribed sphere (fig. S1). The periodic boundary condition of the simulation box must be considered here because the iron cluster tends to spontaneously conform to a cylindrical shape, especially at high pressures and temperatures, probably due to a smaller surface area of the cylinder (considering periodic boundary) compared to sphere. Determination of whether an atom (Mg, Si, O, Pu, and I) resided inside the polyhedron was then done using the random ray crossing algorithm (*54*). More details could be found in (*18*, *19*).

As discussed in (*18*), because of the small size of the system size, one needs to consider the surface effect of iron cluster. The surface effect refers to the potential ambiguity in determining whether the iron atoms on the surface of an iron cluster belong to the silicate cluster or the iron cluster. The irregular shape of the Fe cluster makes it difficult to estimate the number of Fe irons in each phase. In keeping with (*20*), we used valence balance of the silicate phase to consider this effect. By distinguishing whether an atoms is included in the polyhedron, we already know the number of Si, O, Mg, Pu, and I that belong to the iron cluster, $N_{\mathrm{Si}}^{\mathrm{Metal}}$, $N_{O}^{\mathrm{Metal}}$, $N_{\mathrm{Mg}}^{\mathrm{Metal}}$, $N_{\mathrm{Pu}}^{\mathrm{Metal}}$, and $N_{I}^{\mathrm{Metal}}$, and their numbers in the silicate cluster, $N_{\mathrm{Si}}^{\mathrm{Si}\mathrm{licate}}$, $N_{O}^{\mathrm{Silicate}}$, $N_{\mathrm{Mg}}^{\mathrm{Silicate}}$, $N_{\mathrm{Pu}}^{\mathrm{Silicate}}$, and $N_{I}^{\mathrm{Silicate}}$. Using valence balance in the silicate cluster, we obtain

$$N_{\mathrm{Fe}}^{\mathrm{Silicate}}=N_{O}^{\mathrm{Silicate}}-2N_{\mathrm{Si}}^{\mathrm{Silicate}}-N_{\mathrm{Mg}}^{\mathrm{Silicate}}-3/2N_{\mathrm{Pu}}^{\mathrm{Silicate}}$$ (5)

where the valence of Pu in the silicate melt is assumed to be +3 (*55*, *56*) and that of iodine is 0 (*11*). Conservation of the number of atoms means that the number of Fe atoms in the iron cluster is then simply

$$N_{\mathrm{Fe}}^{\mathrm{Metal}}=N_{\mathrm{Fe}}^{\mathrm{Total}}-N_{\mathrm{Fe}}^{\mathrm{Silicate}}$$ (6)

As a result, we know all the numbers of atoms in the two phases at a single snapshot. Here, we use 30,000 to 50,000 snapshots to calculate the average compositions of the liquid iron and silicate melt phases.

### Iodine and plutonium partitioning

Iodine partitioning between silicate melt and iron metal liquid can be described by the reaction in Eq. 1 following (*11*). Although IO^3−^, I_2_, and I^−^ could coexist in the silicate glass (*57*), the exchange coefficient of iodine derived by using the reaction in Eq. 1 could well describe the high *P*-*T* experiment data (*11*). The iodine equilibrium exchange partition coefficient ($K_{D}^{I}$) and equilibrium constant (*K*_I_) are defined as

$$K_{D}^{I}=\frac{X_{I}^{\mathrm{metal}}}{X_{I}^{\mathrm{silicate}}}$$ (7)

$$K_{I}=\frac{\gamma_{I}^{\mathrm{metal}}X_{I}^{\mathrm{metal}}}{\gamma_{I}^{\mathrm{silicate}}X_{I}^{\mathrm{silicate}}}$$ (8)

where *X* denotes the mole fraction and γ is the activity coefficient. The natural logarithm of the equilibrium constant *K*_I_ is the change in Gibbs free energy, which is

$$\ln(K_{I})=-\frac{\text{Δ}H_{I}^{0}-T\text{Δ}S_{I}^{0}+P\text{Δ}V_{i}}{RT}=a_{I}+\frac{b_{I}}{T}+c_{I}\frac{P}{T}$$ (9)

where *P* is pressure (in gigapascal) and *T* is temperature (in Kelvin). The parameters *a*, *b*, and *c* are related to the changes in the standard-state entropy ($\text{Δ}S_{I}^{0}$), enthalpy ($\text{Δ}H_{I}^{0}$), and volume (Δ*V_i_*) of the reaction in Eq. 1. It is noted that equilibrium constants only vary with pressure and temperature and do not depend on *f*o_2_ conditions. We therefore directly regressed the equilibrium constant (instead of the partition coefficient) as a function of pressure and temperature. Once this functional form has been established, the exchange partition coefficient $K_{D}^{I}$ can then be expressed, for any *f*o_2_ condition, as

$$\ln(K_{D}^{I})=\ln(K_{I})-\ln(\gamma_{I}^{\mathrm{metal}})$$ (10)

In the above equation, the γ^silicate^ term is incorporated into the expression of *K*_I_ because activity coefficients in the silicate phase are not strong functions of composition (*58*) following (*11*). The epsilon formalism of (*59*) is used to describe the activity coefficient in the iron-rich metal liquid (the terms of γ_Fe_ and $\gamma_{i}^{0}$ are also incorporated into parameters *a*, *b*, and *c*)

$$\begin{array}{c} \ln(\gamma_{i})=-\varepsilon_{i}^{i}\frac{1873}{T}\ln(1-X_{i})-\sum_{i\neq j}\varepsilon_{i}^{j}\frac{1873}{T}X_{j}\left(1+\frac{\ln(1-X_{j})}{X_{j}}-\frac{1}{1-X_{i}}\right)\\ +\sum_{i\neq j}\varepsilon_{i}^{j}\frac{1873}{T}X_{j}^{2}X_{i}\left(\frac{1}{1-X_{i}}+\frac{1}{1-X_{j}}+\frac{X_{i}}{2{\left(1-X_{i}\right)}^{2}}-1\right) \end{array}$$ (11)

where *i* and *j* are the elements except Fe in iron liquid and $\varepsilon_{i}^{j}$ is the interaction parameter of elements *i* and *j* at the standard temperature of 1873 K. Under the consideration of the concentration of elements in the metal liquid, $\varepsilon_{I}^{O}$, $\varepsilon_{I}^{S}$, and $\varepsilon_{I}^{C}$ are included in the expression of $\gamma_{I}^{\mathrm{metal}}$. All parameters ($i.e.,a_{I},b_{I},c_{I},\varepsilon_{I}^{O}$, $\varepsilon_{I}^{S}$, and $\varepsilon_{I}^{C}$) were then fitted to exchange coefficients of first-principles calculation and LH-DAC experimental (*11*) data by the least-squares method and yielded *a*_I_ = 1.34 ± 1.51, *b*_I_ = − (13.4 ± 4.8) × 10^3^, *c*_I_ = 131 ± 48, $\varepsilon_{I}^{O}=-9.13\pm 4.16$, and $\varepsilon_{I}^{S}=-5.71\pm 2.29$. Because the model with or without $\varepsilon_{I}^{C}$ fits the data equally well based on *F* test (probability value: *P* > 0.05), $\varepsilon_{I}^{C}$ was set to 0 in this work. Although the uncertainty of *a*_I_ is higher than its value, the *a*_I_ term cannot be neglected because it account for the entropy change of reaction (*58*).

Plutonium partitioning between silicate melt and iron metal liquid can be described as a dissociation reaction in Eq. 2. As trivalent Pu is verified to be enriched in silicate minerals and glass at high temperature (*55*, *56*), we assume Pu to behave similarly in metal-silicate partitioning situations (*25*). The plutonium equilibrium exchange partition coefficient ($K_{D}^{\mathrm{Pu}}$) and equilibrium constant (*K*_Pu_) are defined as

$$K_{D}^{\mathrm{Pu}}=\frac{X_{\mathrm{Pu}}^{\mathrm{metal}}{\left(X_{O}^{\mathrm{metal}}\right)}^{\frac{3}{2}}}{X_{\mathrm{Pu}O_{\frac{3}{2}}}^{\mathrm{silicate}}}$$ (12)

$$K_{\mathrm{Pu}}=\frac{\gamma_{\mathrm{Pu}}^{\mathrm{metal}}X_{\mathrm{Pu}}^{\mathrm{metal}}{\left(\gamma_{O}^{\mathrm{metal}}X_{O}^{\mathrm{metal}}\right)}^{\frac{3}{2}}}{\gamma_{\mathrm{Pu}O_{\frac{3}{2}}}^{\mathrm{silicate}}X_{\mathrm{Pu}O_{\frac{3}{2}}}^{\mathrm{silicate}}}$$ (13)

Because there are only three FPMD data points for Pu and no experimental data with detailed composition data to calculate $K_{D}^{\mathrm{Pu}}$ (*17*), too many parameters would result in overfitting, and we therefore assumed

$$\ln(K_{D}^{\mathrm{Pu}})=\ln(K_{\mathrm{Pu}})=a_{\mathrm{Pu}}+\frac{b_{\mathrm{Pu}}}{T}$$ (14)

Parameters *a*_Pu_ and *b*_Pu_ were fitted to exchange coefficients by the least-squares method and yielded *a*_Pu_ = 11.1 ± 1.1 and *b*_Pu_ = − (56.8 ± 3.5) × 10^3^.

In theory, because equilibrium constants do not depend on *f*o_2_, experiments and FPMD simulations could be performed at any *f*o_2_ conditions and still be used in core formation modeling. In practice and to avoid extrapolation, it is preferable to run experiments and FPMD simulations under conditions relevant to the core formation modeling. This is why our simulations were conducted at *f*o_2_ values between IW-1.9 and IW-2.9. These values cover the range of values used in typical core formation models (*1*, *22*, *23*) and encompass our preferred accretion model whereby Earth mainly accreted from differentiated planetesimals thought to be very reduced. That range is similar to the conditions of the more oxidized piston cylinder experiment (IW-1.9) in (*11*) and is only ~1 to 2 log units below (i.e., more reduced than) those of the DAC experiment (IW-0.5 to IW-2.0). Overall, all available data (FPMD and experiments) focused on the quite reducing conditions thought to have been relevant to core formation on Earth [IW-1.5 to IW-2.5; e.g., (*23*, *28*)]. Thus, when possible (i.e., for iodine), all data are considered to build the equilibrium constant fit as function of pressure and temperature.

A final quality control on our simulations was performed by calculating the major element equilibrium constants obtained in our FPMD simulations. As shown in fig. S10, the equilibrium constants for Si, O, and Mg from our new FPMD data are fully consistent with previous FPMD data and experimental data obtained at *f*o_2_ ranging from IW-0.4 to IW-4.0. This excellent agreement further demonstrates (i) the adequacy of our FPMD method and (ii) the feasibility of regressing equilibrium constant to obtain partition coefficients, even with a limited number of simulations.

### Core formation modeling

Core formation was modeled as a multistage process during Earth’s accretion (*22*, *60*). In the fiducial model of this work, Earth was accreted to its present mass in 0.1% increments (i.e., 1000 steps). The metal fraction in the impactor was set to 0.325 in the fiducial model. Each impact was assumed to generate a magma ocean at the top of the proto-Earth, within which the metal in the impactor fully equilibrated with the entire magma ocean (including the silicate in the impactor) under the pressure and temperature of the base of the magma ocean (*1*, *22*). After equilibrium, the equilibrated liquid metal was assumed to descend rapidly to the core through the solid mantle without further equilibration (*60*) and the equilibrated magma ocean mixed with the solid mantle to form a new mantle (*1*). The above assumptions, while necessarily simplistic, were made as extreme examples to enable the expression of the largest core formation–induced I/Pu fractionation. The rationale being that if even this scenario cannot explain the I/Pu contrast between MORBs and OIBs, then more realistic scenarios (e.g., invoking partial equilibration) would not either.

During this incremental accretion process, the pressure at the base of the magma ocean increased with the fraction of mass accreted (*f*), following (*22*)

$$P=P_{\mathrm{fin}}\times f^{\frac{2}{3}}$$ (15)

where *P*_fin_ is the equilibrium pressure of the magma ocean at the last accretion. Using this equilibrium pressure, the temperature was determined on the basis of a cold liquidus geotherm constructed through the melting of chondritic mantle (*21*) in the fiducial model. The fraction of mantle mass involved in the magma ocean (*f*_mo_) was parameterized as a function of *P*_fin_ following (*1*)

$$f_{\mathrm{mo}}=\frac{r_{\mathrm{earth}}^{3}-{\left[r_{\mathrm{earth}}-(r_{\mathrm{earth}}-r_{\mathrm{core}})\times \frac{P_{\mathrm{fin}}}{P_{\mathrm{CMB}}}\right]}^{3}}{r_{\mathrm{earth}}^{3}-r_{\mathrm{core}}^{3}}$$ (16)

where the radius of Earth (*r*_earth_) is 6371 km, the radius of Earth’s core (*r*_core_) is 3485 km, and the pressure at CMB (*P*_CMB_) is 135 GPa. In the fiducial model, *P*_fin_ is assumed to be 60 GPa.

During the multistage core formation process, the distribution of elements was calculated by mass balance using their partition coefficients. The concentrations of O, Si, Co, Cr, V, and Ni were first derived at each step. The partition coefficient of these elements were taken from (*61*, *62*) following (*22*). In each accreting building block, the bulk concentrations of these elements (except for O and Fe) relative to Al were those of the bulk Earth’s composition assuming 5 wt % of Si in core (*63*), following (*1*). In principle, the *f*o_2_ of Earth would vary as accretion proceeds because of loss of O to space (*64*), which would affect the size of Earth’s core (*29*). Because it is hard to precisely evaluate the O escape to space during accretion, to test how *f*o_2_ affects the accretion process, we assume that a constant core mass fraction during Earth’s accretion and the concentration of O and Fe were instead controlled by predetermined evolution scenarios of the FeO content in the magma ocean. These evolution histories were constructed by linear interpolation between different meteorites as a starting point and present Earth’s mantle as an ending point [figure 1 in (*22*)]. The fiducial model used the FeO in high-iron enstatite (EH) chondrite as the starting point [path 2 of figure 1 in (*22*)]. This fiducial model illustrates a scenario in which Earth started accretion from a plausible reduced redox condition but does not mean that Earth formed through accretion of EH chondrites. Then, the distribution of Pu and I were determined on the basis of the pressure, temperature, and metal compositions for each step using Eqs. 3 and 4 and their activity coefficients.

Besides the fiducial model, a series of simulations were performed in which key input parameters were varied to test the sensitivity of these parameters on the simulation outcomes. These runs tested different (i) *P*_fin_ values (60, 70, and 80 GPa) that determine the equilibrium pressure, (ii) mantle liquidus geotherm [hot (*26*) and cold (*21*)] that determines the equilibrium temperature, and (iii) evolution scenario of FeO content of the magma ocean [paths 2, 9, 10, 11, 12, 13, and 14 of figure 1 in (*22*)], ensuring that the *f*o_2_ conditions are varied. A special accretion scenario of equilibrium pressure was also constructed to verify whether high-pressure episodes in the middle stages of accretion could efficiently fractionate I and Pu. In this scenario, equilibrium pressures during the first 50% of the accretion was the pressure at the CMB of the proto-Earth (*P*_fin_ = 135 GPa), and the pressure during the last 50% accretion linearly decreased from the highest pressure (85 GPa) to a final equilibrium pressure (40 GPa). We also tested whether the change of core size would affect I/Pu fractionation during accretion process. Heterogeneous volatile accretion simulations were performed by multiplying iodine concentration of building block materials in phase 2, while Pu concentration was assumed to remain constant throughout Earth’s accretion.

### Meteoric Xe analysis

Xenon has nine isotopes (i.e., ^124^Xe, ^126^Xe, ^128^Xe, ^129^Xe, ^130^Xe, ^131^Xe, ^132^Xe, ^134^Xe, and ^136^Xe) whose relative abundances represent a mixture of six components (see table S3): (i) spallogenic xenon (noted here sp), (ii) initial or primordial xenon (init) that can be represented by solar wind or Phase-Q, (iii) atmospheric xenon (air), (iv) uranium-fission xenon (U), (v) plutonium-fission xenon (Pu), and (vi) iodine-decay xenon. Except for the iodine-decay component, ^132^Xe is present in all other components. So, we can normalize to ^132^Xe (avoiding issues from divisions by zero) and decompose the isotopic composition in a sample as the sum of contributions from the first five components as

$$\begin{array}{c} X_{\mathrm{sp}}{\left(\frac{{}^{i}\mathrm{Xe}}{{}^{132}\mathrm{Xe}}\right)}_{\mathrm{sp}}+X_{\mathrm{init}}{\left(\frac{{}^{i}\mathrm{Xe}}{{}^{132}\mathrm{Xe}}\right)}_{\mathrm{init}}+X_{\mathrm{air}}{\left(\frac{{}^{i}\mathrm{Xe}}{{}^{132}\mathrm{Xe}}\right)}_{\mathrm{air}}+X_{\mathrm{Pu}}{\left(\frac{{}^{i}\mathrm{Xe}}{{}^{132}\mathrm{Xe}\;}\right)}_{\mathrm{Pu}}+X_{U}{\left(\frac{{}^{i}\mathrm{Xe}}{{}^{132}\mathrm{Xe}}\right)}_{U}\\ ={\left(\frac{{}^{i}\mathrm{Xe}}{{}^{132}\mathrm{Xe}}\right)}_{\mathrm{meteorite}} \end{array}$$ (17)

where *i* is 124, 126, 128, 130, 131, 134, or 136 and *X* is the molar proportion of ^132^Xe in the sample from each component. For the solution of *X* to be physically relevant, the following additional constraints are considered

$$X_{\mathrm{sp}}+X_{\mathrm{init}}+X_{\mathrm{air}}+X_{\mathrm{Pu}}+X_{U}=1$$ (18)

$$\begin{array}{c} X_{\mathrm{sp}}{\left(\frac{{}^{129}\mathrm{Xe}}{{}^{132}\mathrm{Xe}}\right)}_{\mathrm{sp}}+X_{\mathrm{init}}{\left(\frac{{}^{129}\mathrm{Xe}}{{}^{132}\mathrm{Xe}}\right)}_{\mathrm{init}}+X_{\mathrm{air}}{\left(\frac{{}^{129}\mathrm{Xe}}{{}^{132}\mathrm{Xe}}\right)}_{\mathrm{air}}+X_{\mathrm{Pu}}{\left(\frac{{}^{129}\mathrm{Xe}}{{}^{132}\mathrm{Xe}}\right)}_{\mathrm{Pu}}+X_{U}{\left(\frac{{}^{129}\mathrm{Xe}}{{}^{132}\mathrm{Xe}}\right)}_{U}\\ \leq {\left(\frac{{}^{129}\mathrm{Xe}}{{}^{132}\mathrm{Xe}}\right)}_{\mathrm{meteorite}} \end{array}$$ (19)

$$\mathrm{and}\;0\leq X_{j}\leq 1$$ (20)

where *j* is sp, init, air, Pu, and U, respectively. There are five unknowns and seven equations. The linear least-squares solutions to this system of equation with constraints were analytically derived using the method of Lagrange multipliers. Following (*9*), each endmember and sample isotope ratio was weighed by normalization to the 1σ derivations on the meteorite compositions. Once the solution of the least-squares decomposition was obtained, the ^129^Xe*/^136^Xe*_Pu_ for a given sample was calculated as follows

$$\frac{{}^{129}Xe^{*}}{{}^{136}Xe_{\mathrm{Pu}}^{*}}=\frac{{\left(\frac{{}^{129}Xe}{{}^{132}Xe}\right)}_{\mathrm{meteorite}}-\sum_{j}X_{j}{\left(\frac{{}^{129}Xe}{{}^{132}Xe}\right)}_{j}\;}{X_{\mathrm{Pu}}{\left(\frac{{}^{136}Xe}{{}^{132}Xe}\right)}_{\mathrm{Pu}}}$$ (21)

A Monte Carlo method was used to propagate the uncertainties in mixing proportions for each endmember. Specifically, 10^5^ Monte Carlo simulations are applied for each sample by repeatedly sampling its compositions based on isotopic values and uncertainties. There are 9 chondrites and 10 achondrites data used in this work (see tables S3 to S5), and this exercise points to the need for further high-precision Xe isotope investigation of meteorites.

## Supplementary Materials

### This PDF file includes:

Figs. S1 to S14 Tables S1 to S5 References
