# Supplementary material for: I/Pu reveals Earth mainly accreted from volatile-poor differentiated planetesimals
Source: Sci Adv. 2023 Jul 5;9(27):eadg9213. doi: 10.1126/sciadv.adg9213 (PMC10321745; doi:10.1126/sciadv.adg9213)
Supplement: Supplementary file 1 — Figs. S1 to S14 Tables S1 to S5 References [file sciadv.adg9213_sm.pdf]

Science Advances
AAAS

Supplementary Materials for

I/Pu reveals Earth mainly accreted from volatile-poor differentiated planetesimals

Weiyi Liu *et al.*

Corresponding author: Weiyi Liu, weiyiliu@caltech.edu

Sci. Adv. **9**, eadg9213 (2023)
DOI: 10.1126/sciadv.adg9213

This PDF file includes:

Figs. S1 to S14
Tables S1 to S5
References

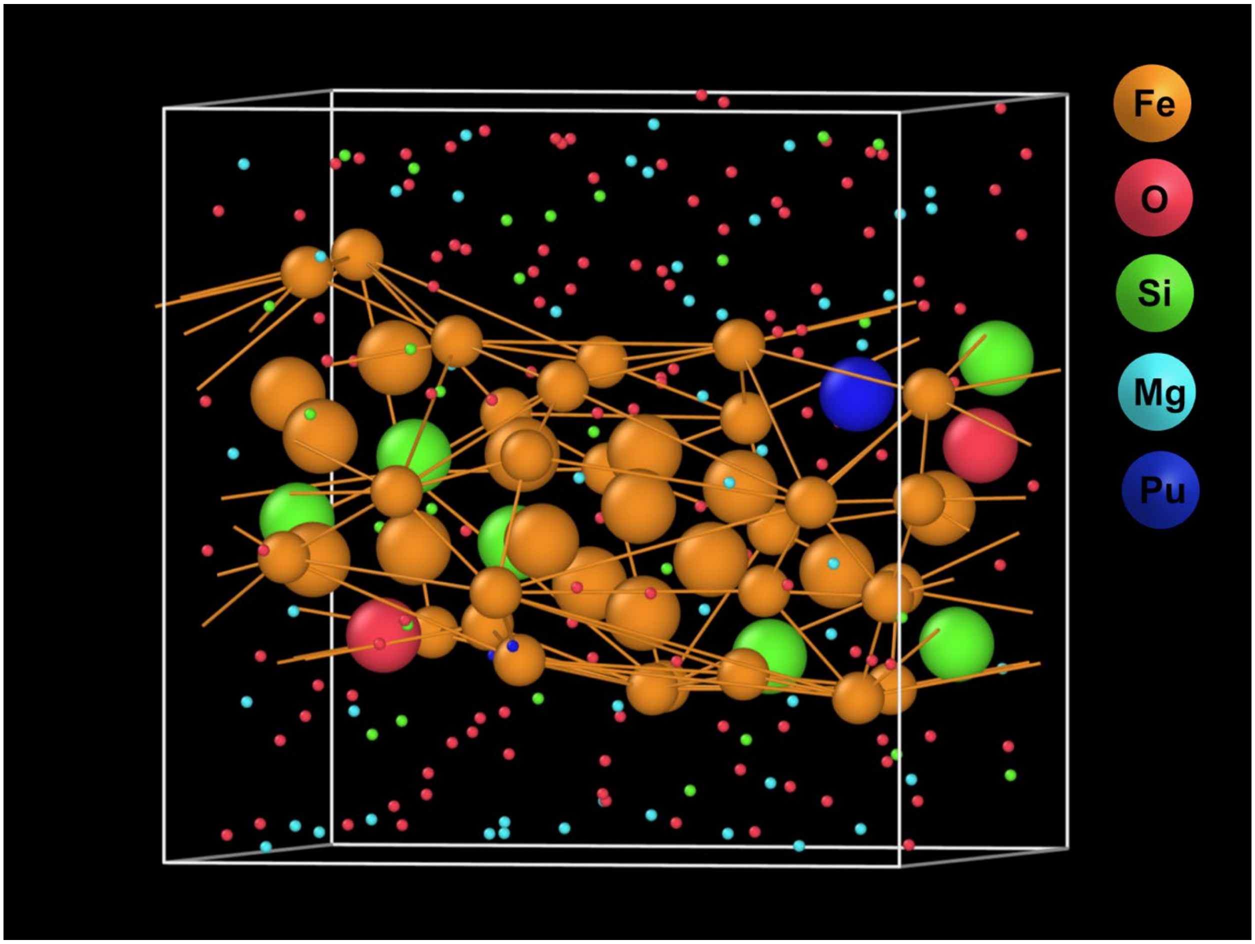

Figure S1. Snapshot of atomic configuration in the simulation cell. The iron cluster (large atom symbols) at center marks the liquid metal phase domain and the surrounding area (small atom symbols) is the silicate melt domain. The orange bonds illustrate the surface of the constructed alpha shape of the Fe cluster considering the periodic conditions of the simulation cell. Some Si, O and Pu atoms (large atom marks) are enclosed in the alpha shape.

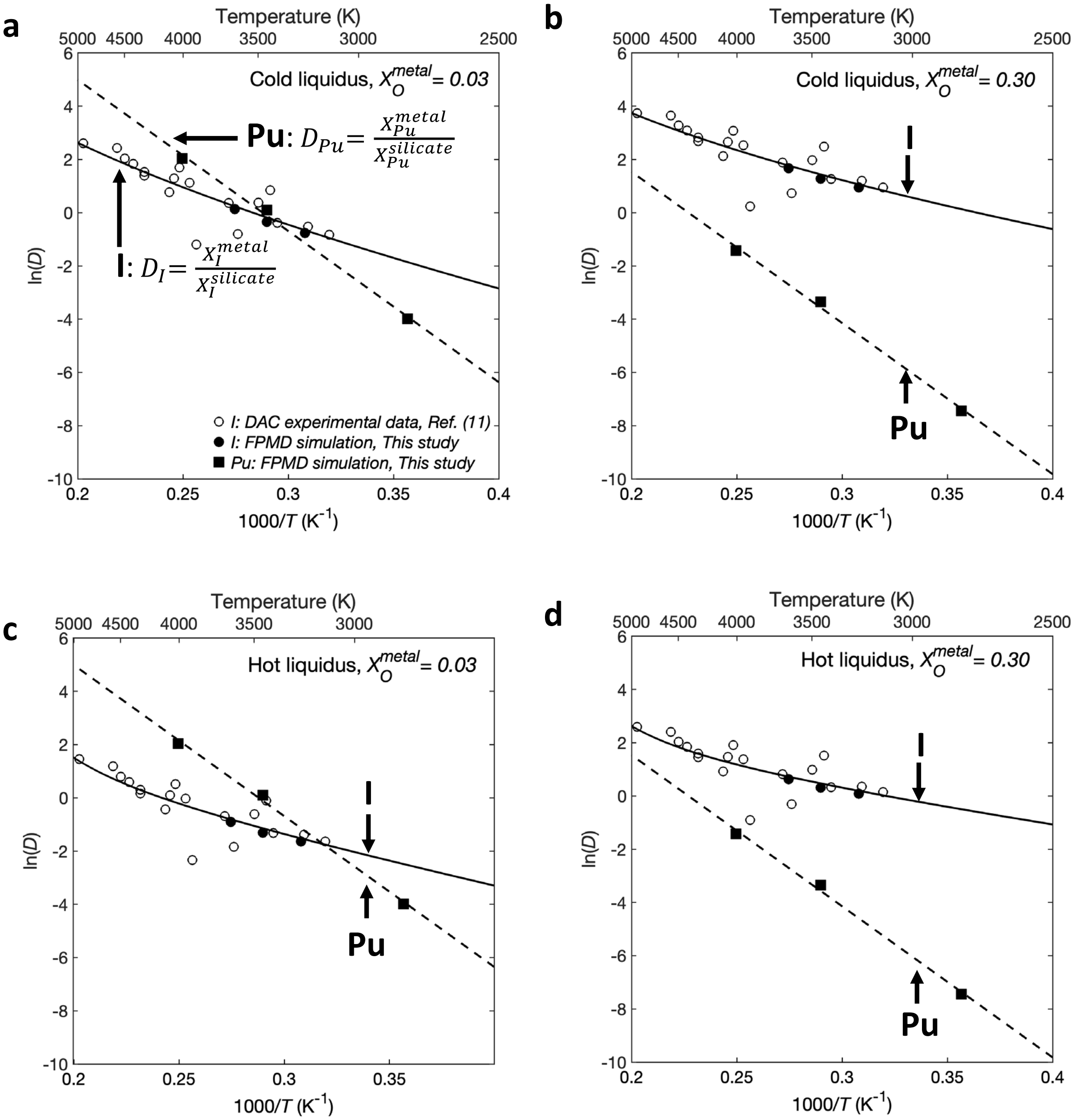

Figure S2. Metal-silicate partition coefficients for iodine and plutonium as a function of temperature at different P-T-X_O^{metal} conditions. Partition coefficients ($D = X^{metal} / X^{silicate}$) for iodine (solid lines) and plutonium (dashed lines) are derived from equilibrium constants for given **P-T-X_O^{metal}** conditions. The pressures and temperatures are connected by either a cold liquidus geotherm (a, b) using chondritic mantle composition (*21*), or and a hot liquidus geotherm (c, d) constructed by peridotite (*26*) which was used in ref. (*11*). X_O^{metal} is set to be 3 mol% (a, c) and 30 mol% (b, d). The 3 mol% and 30 mol% oxygen in metal correspond to ~1 wt% and 10 wt%, respectively, and cover the range of oxygen concentration consistent with the seismic observations

(*22*, *65*). Measured D values from this study and previous experimental study (*11*) are corrected to the given X_O^{metal} and the mantle liquidus geotherm according to temperature. It is noted that X_O^{metal} would change with pressure and temperature during core formation processes. In the more realistic cases, the partition coefficients would evolve with the mass accreted to the Earth as shown in Fig. S7.

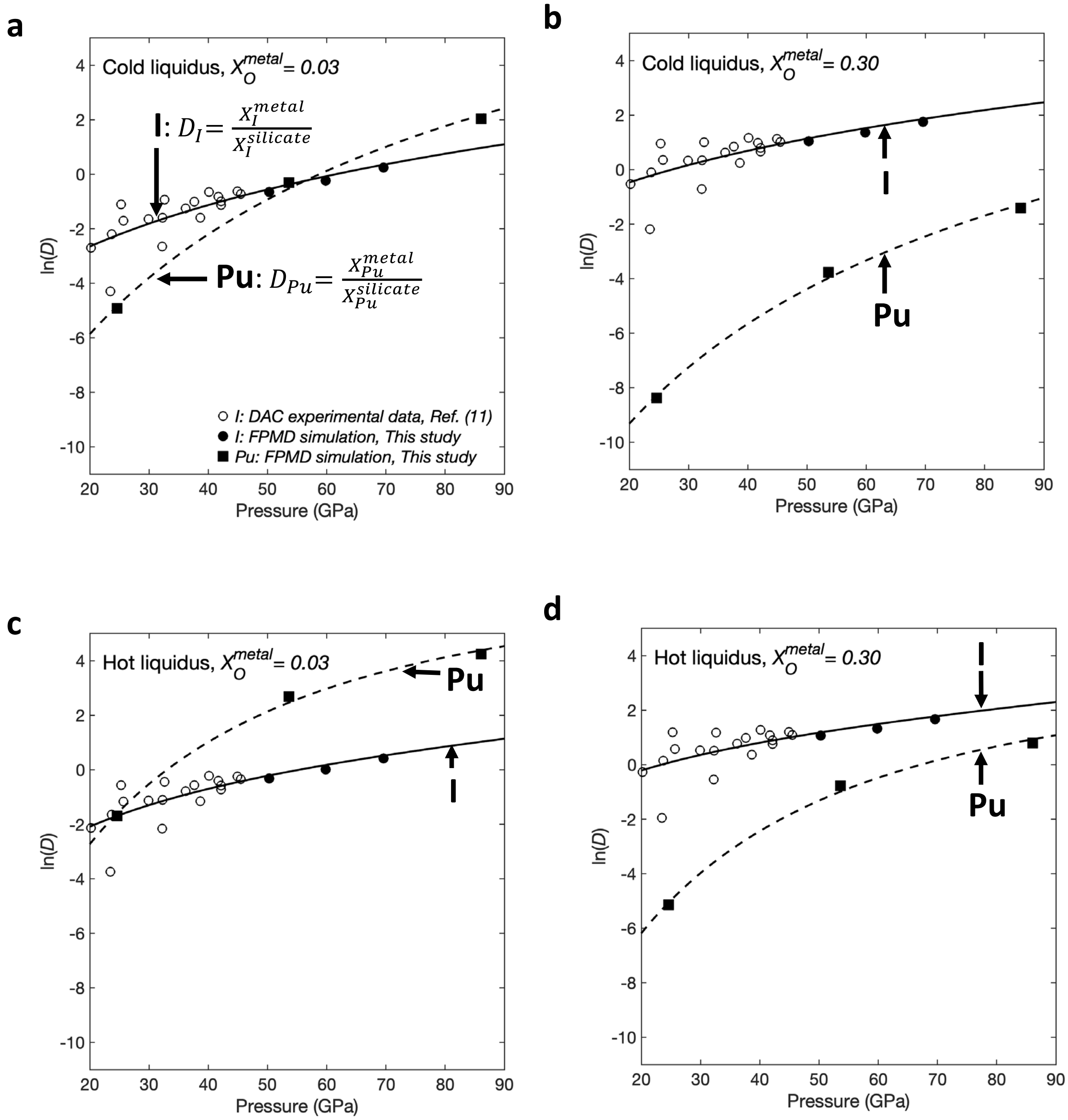

Figure S3. Metal-silicate partition coefficients for iodine and plutonium as a function of pressure at different P-T-X_O^{metal} conditions. Symbols, notations and assumptions as on Figure S2. Measured D values from this study and previous experimental study (*11*) are corrected to the given X_O^{metal} and the mantle liquidus geotherm according to pressure.

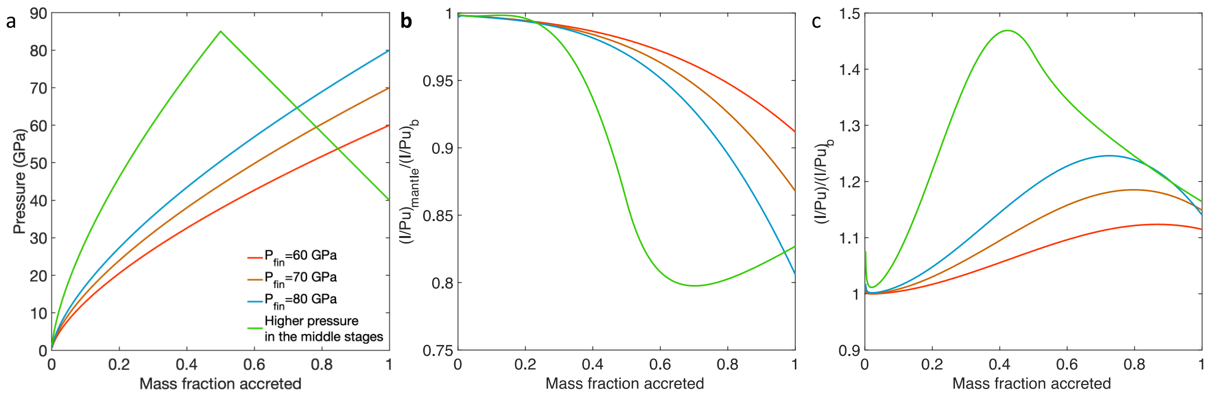

Figure S4. Pressure and temperature sensitivity tests for homogeneous volatile accretion scenarios. Model runs assume a reduced oxygen fugacity condition in which the FeO concentration in the magma ocean linearly increase with mass fraction accreted to the Earth from 0.6 mol% to 5.8 mol% (Path 2 of Fig.1 in ref. (*22*)). Materials accreted to the Earth have identical I/Pu ratios during all stages of accretion. (a) Equilibrium pressures as a function of mass fraction accreted, for different accretion scenarios. The red, yellow and blue lines are 3 examples of incrementally increasing equilibrium pressure with different final pressures (P_{fin}). The green line is an example of accretion scenario in which equilibrium pressures during the first 50% of Earth's accretion is the pressure at the CMB of the proto-Earth and the pressure during the last 50% of Earth's accretion linearly decreases from 85 GPa to 40 GPa. (b-c) Variations of the mantle I/Pu ratio (normalized to the I/Pu ratio of the bulk accretion materials) as a function of the mass fraction accreted assuming an equilibrium temperature that follows (b) the cold liquidus geotherm constructed through the melting of chondritic mantle (*21*), or (c) the hot liquidus geotherm based on the melting profiles of peridotite(*26*). Curves color coded as in (a).

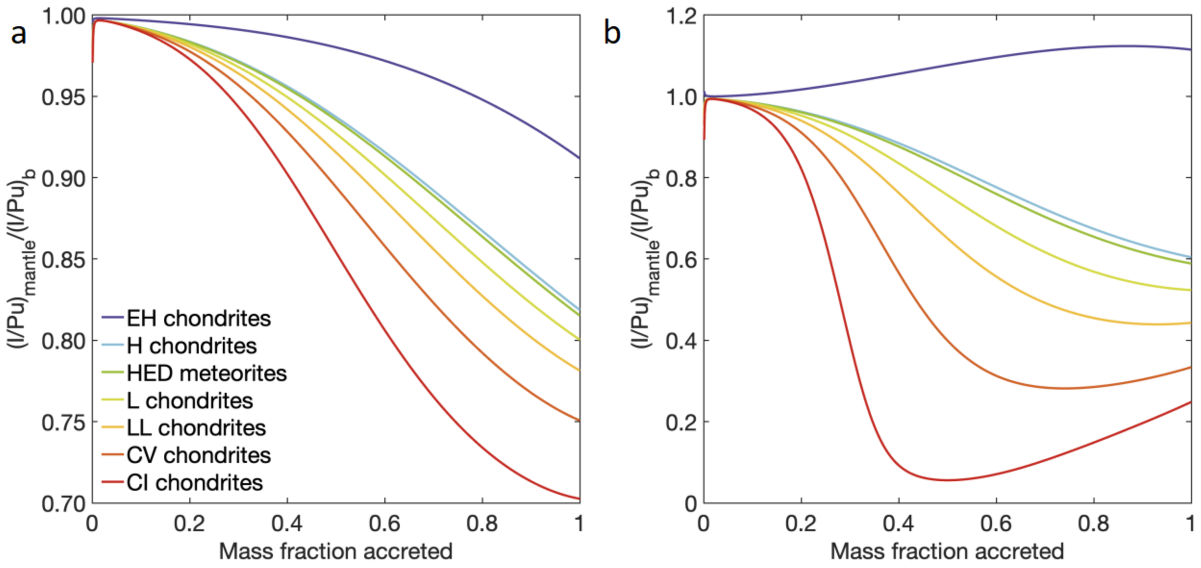

Figure S5. Variations of the mantle I/Pu ratio as a function of the mass fraction accreted to the Earth under homogeneous volatile accretion scenarios for different *f*O_2 conditions and equilibrium T. In all runs the equilibrium incrementally increases to a final value of 60 GPa, and materials accreted to the Earth have identical I/Pu ratios during all stages of accretion. Colors denote the predetermined oxygen fugacity evolution scenarios of the FeO concentration in the magma ocean, which are constructed by linear interpolation between different meteorites compositions (*i.e.*, EH chondrites (initial redox: IW-4.5), H chondrites (IW-1.4), HED chondrites (IW-1.3), L chondrites (IW-1.2), LL chondrites (IW-1.0), CV chondrites (IW-0.8), and CI chondrites (IW-0.6)) as a starting point and the present Earth's mantle as an ending point (Paths 2, 9, 10, 11, 12, 13 and 14 of Fig.1 in ref. (*22*)). These models illustrate scenarios in which the Earth started accretion under the entire plausible range of redox conditions, but does not mean that the Earth formed by accretion of the type of meteorites in the legend. (a-b) Variations of the mantle I/Pu ratio (normalized to the I/Pu ratio of the bulk accretion materials) for different evolution scenario of FeO content in the magma ocean and an equilibrium temperature following (a) the cold liquidus geotherm (*21*), and (b) the hot liquidus geotherm (*26*). Though the composition of peridotite cannot reflect the bulk mantle, its melting profile could represent the upper-limit of equilibrium temperatures during core formation. It is noted that the change of core size would also affect the oxygen fugacity during accretion process. However, our result shows that core size would not have notable impact on I/Pu differentiation, and thus the change of impactor core fraction during accretion does not affect our result (Fig. S11).

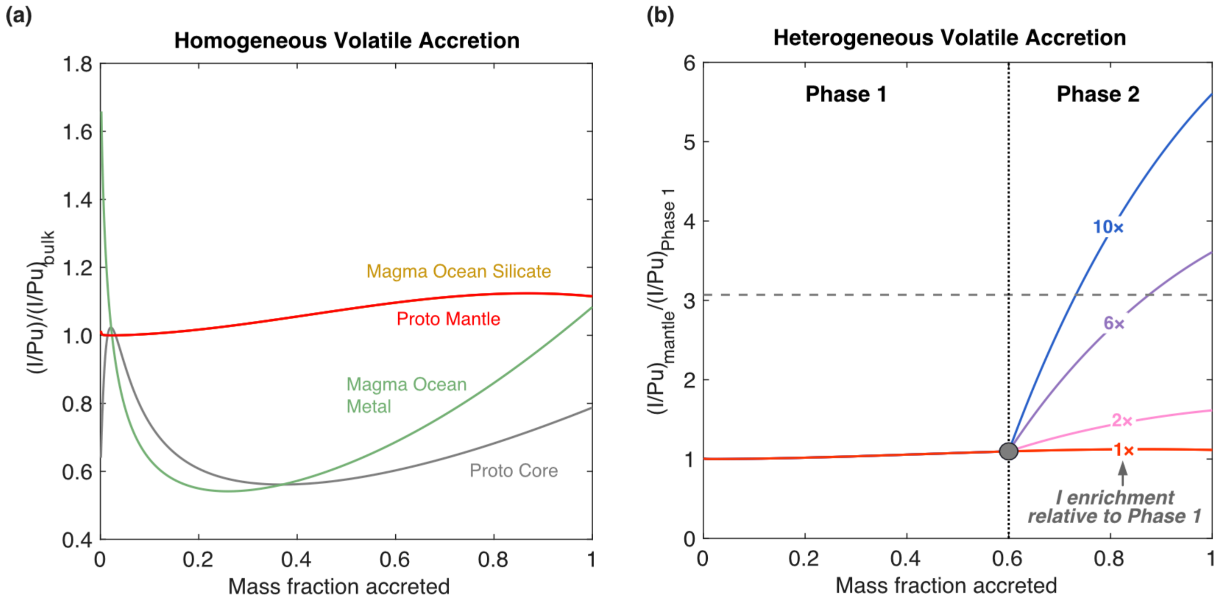

Figure S6. Variations of I/Pu ratio with mass fraction accreted to the Earth under homogeneous and heterogeneous accretion scenarios in an equilibrium temperature that follows hot liquidus geotherm. Same as Fig. 2 in the main text, except simulations are made under an equilibrium temperature that follows the hot liquidus geotherm. (a) Though the composition of peridotite cannot reflect the bulk mantle, its melting profile could represent the upper-limit of equilibrium temperatures during core formation. The lowermost I/Pu ratio of magma ocean metal (0.55), which is twice lower compared to the mantle during the late stages of accretion (~1.1), could be treated as the lower limit for the I/Pu ratio in the liquid metal droplet that never settled to the Earth's core. (b) In the later stages of accretion (Phase 2), iodine content in the material accreted to the Earth are higher (see enrichment factor on each colored curve) than during the earlier stages of accretion (Phase 1).

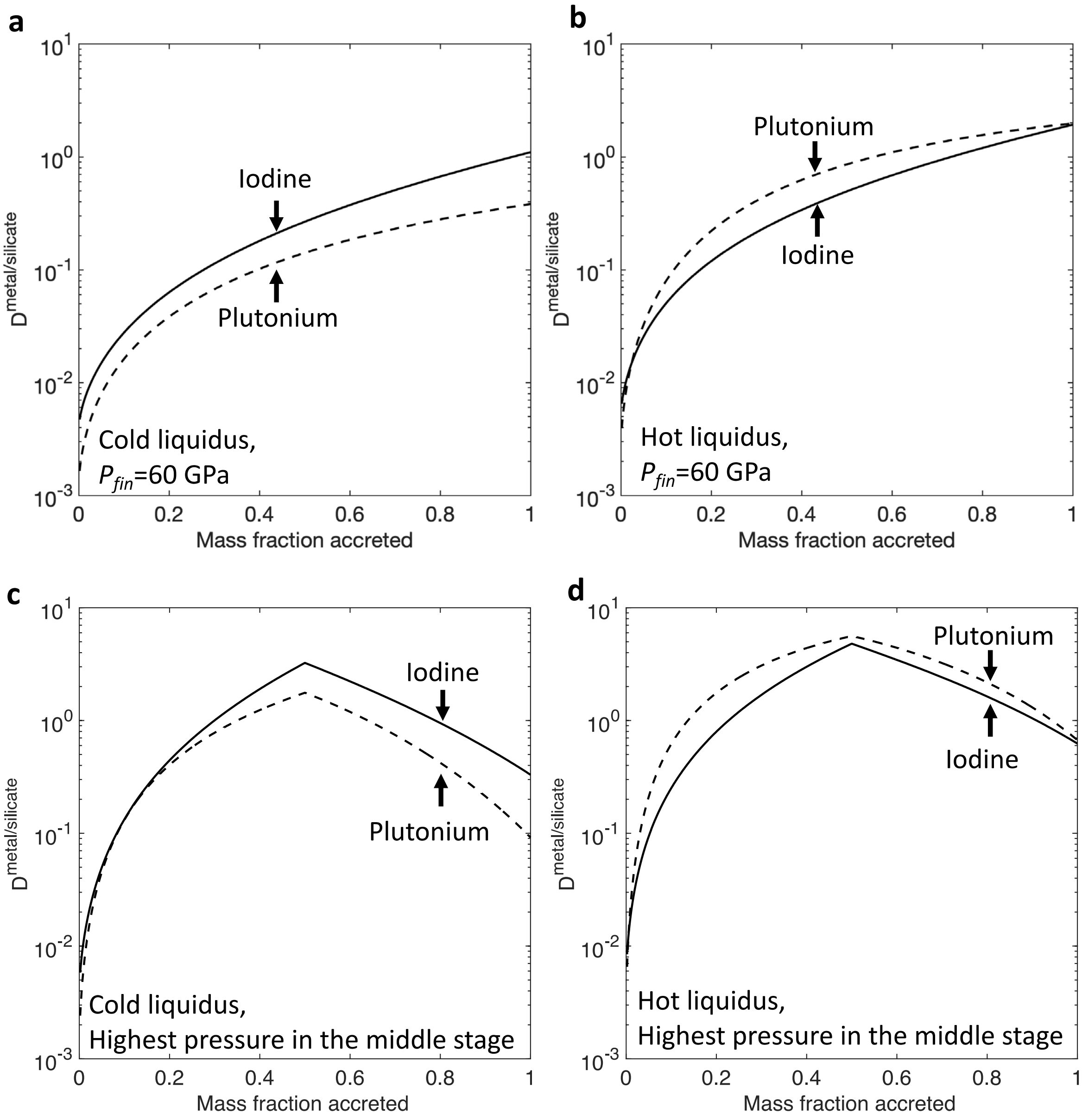

Figure S7. Variations of I and Pu partitioning between silicate melt and metal liquid as a function of the mass accreted to the Earth. (a-b) The simulations are made under the fiducial model as described in the Methods. (c-d) The simulations are made assuming an episode of high-pressure equilibration in the middle of Earth's accretion (green line in Fig S4.a). The pressures and temperatures during accretion processes are connected following (a,c) the cold liquidus geotherm (*21*) and (b,d) the hot liquidus geotherm (*26*). The solid lines are I partition coefficients, and the dashed lines are Pu partition coefficients. Both I and Pu become less lithophile (D values approaches 1) as equilibration pressure increasing, and even become moderately siderophile at highest equilibration pressure (D values above 1).

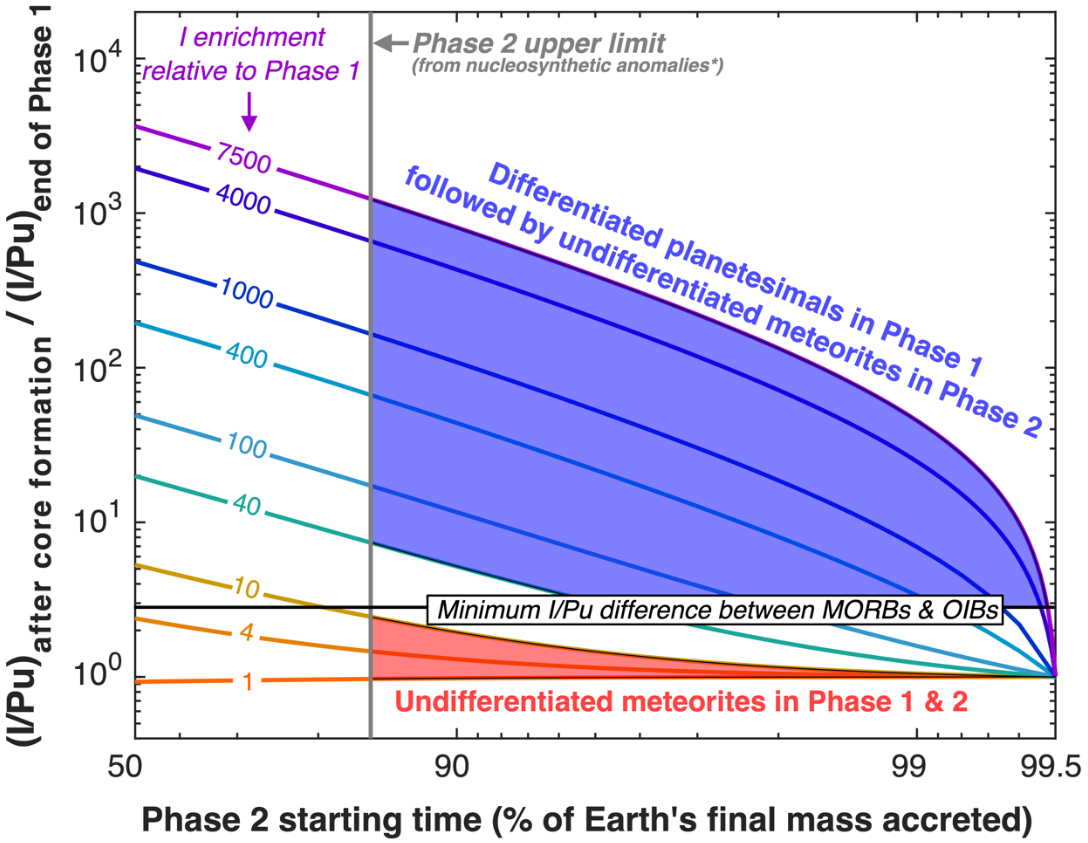

Figure S8. Mantle's I/Pu ratio right after core formation processes (normalized to the mantle I/Pu at the end of Phase 1) as a function of Phase 2 starting time. The mantle's I/Pu ratio after core formation could be treated as the endmember scenario in which late veneer happened after ^{129}I extinction and cannot deliver any ^{129}Xe to the Earth. The simulations conditions and the meaning of curves are same as that in Figure 3.

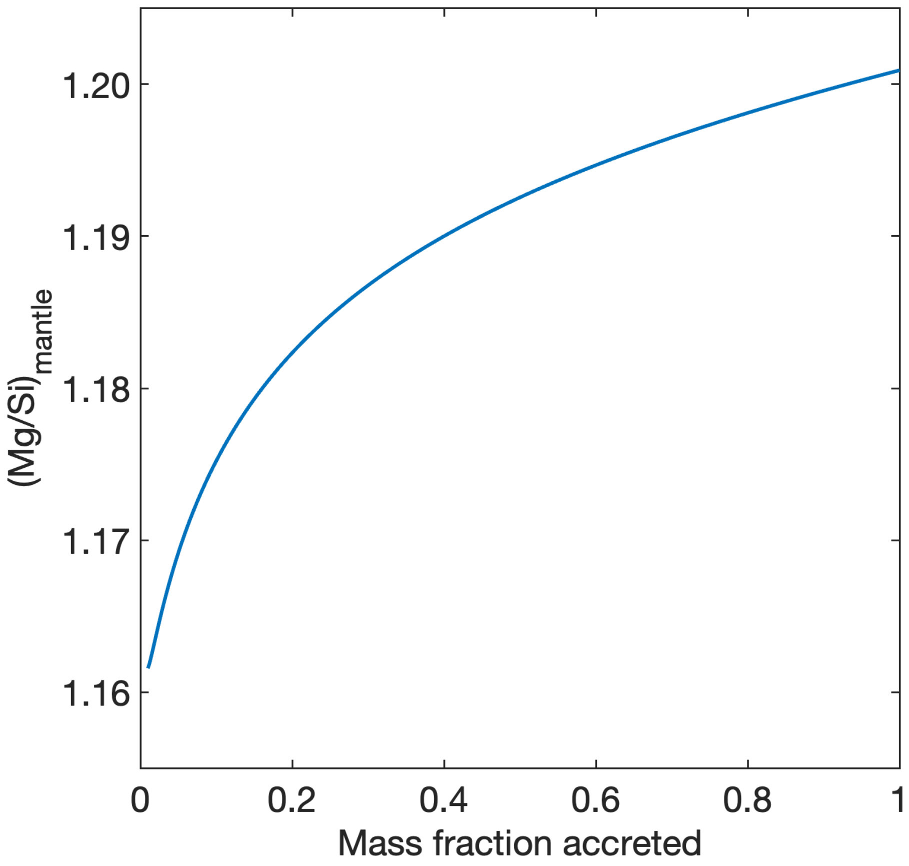

Figure S9. Variations of mantle Mg/Si ratio as a function of the mass fraction accreted to the Earth. The simulation is made under the fiducial model as described in Methods. Si enters the metallic core at high P-T equilibration, and thus results in a high Mg/Si mantle during the late stage of accretion.

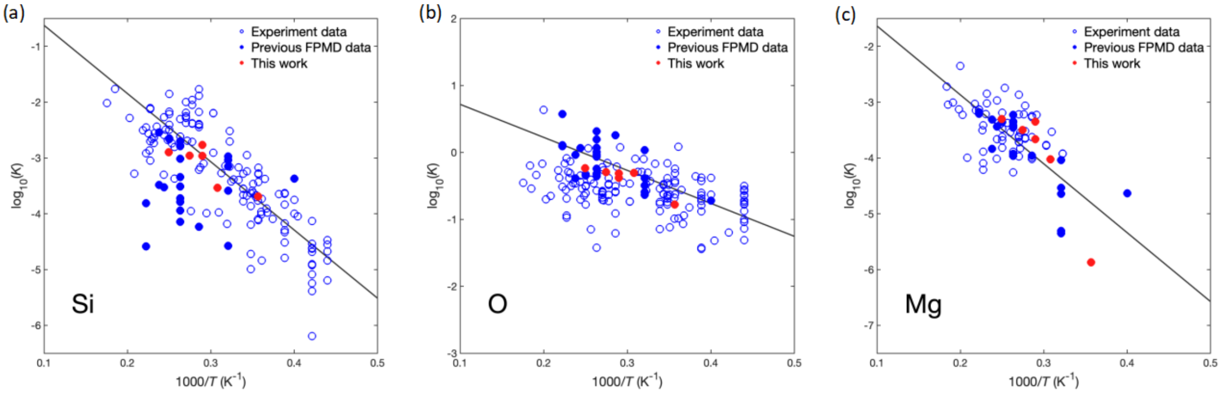

Figure S10. Comparison of equilibrium constants from new FPMD simulations with those from previous experiment and FPMD simulations. The central thick straight lines are the fitting equations of equilibrium constant in ref. (*20*). The blue open circles are experimental data (*11*, *24*, *33*, *58*, *60*, *61*, *66–80*), the blue filled circles are pervious FPMD data (*20*), and the red filled circles are new FPMD data from present study. The equilibrium constants are derived by using exchange coefficients and activity coefficients with $\epsilon_{Si}^{O} = -8.3$, $\epsilon_{O}^{O} = -5.8$ and $\epsilon_{Mg}^{O} = -16.4$. See ref. (*20*) for more details. (a) Equilibrium constant of reaction $SiO_2^{Sil} + 2Fe^{Met} = 2FeO^{Sil} + Si^{Met}$. (b) Equilibrium constant of reaction $FeO^{Sil} = Fe^{Met} + O^{Met}$. (c) Equilibrium constant of reation $MgO^{Sil} = Mg^{Met} + O^{Met}$.

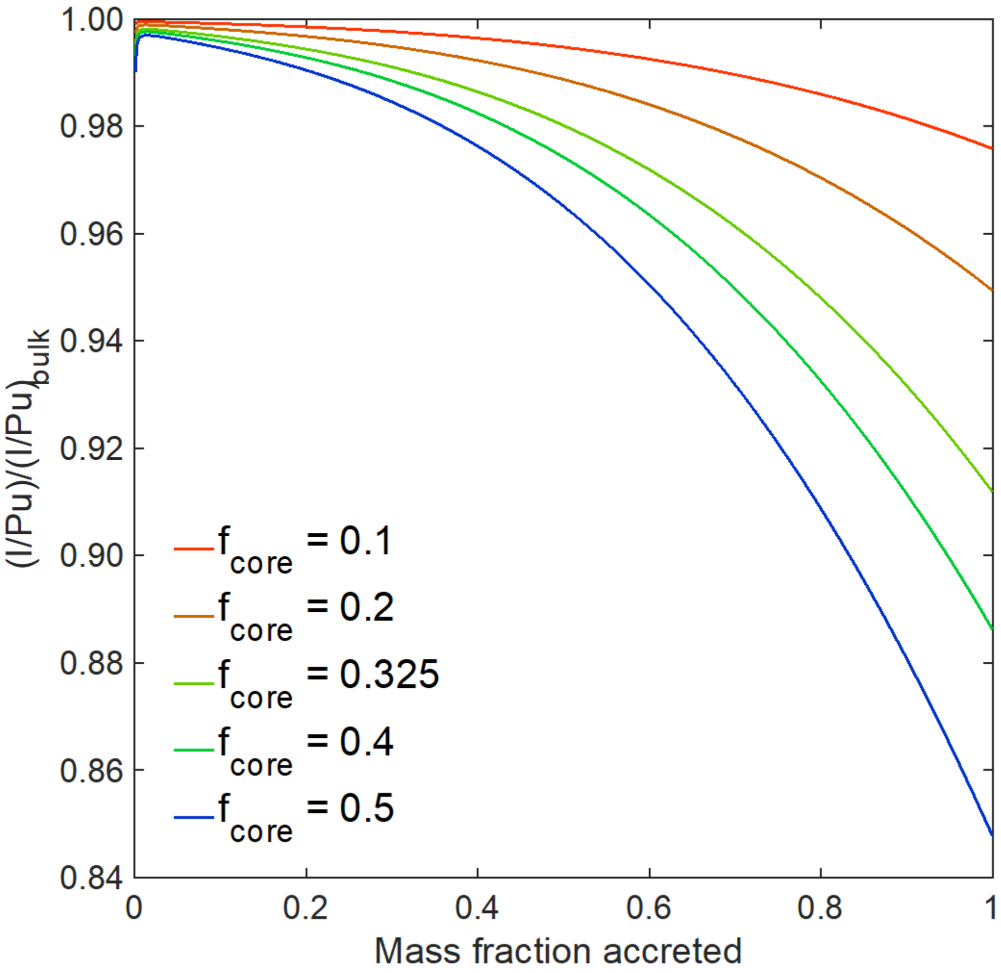

Figure S11. Variations of the mantle I/Pu ratio as a function of the mass fraction accreted to the Earth under homogeneous volatile accretion scenarios for different metal fraction in the impactor (f_{core}). The simulations are made based on the fiducial model as described in the Methods. This figure shows that changes in the $\mathbf{f_{core}}$ of impactors and the accreting Earth would only marginally impact the I/Pu ratio during differentiation (less than 20%). As such, changes in impactor core fraction during accretion do not affect the results presented in this work.

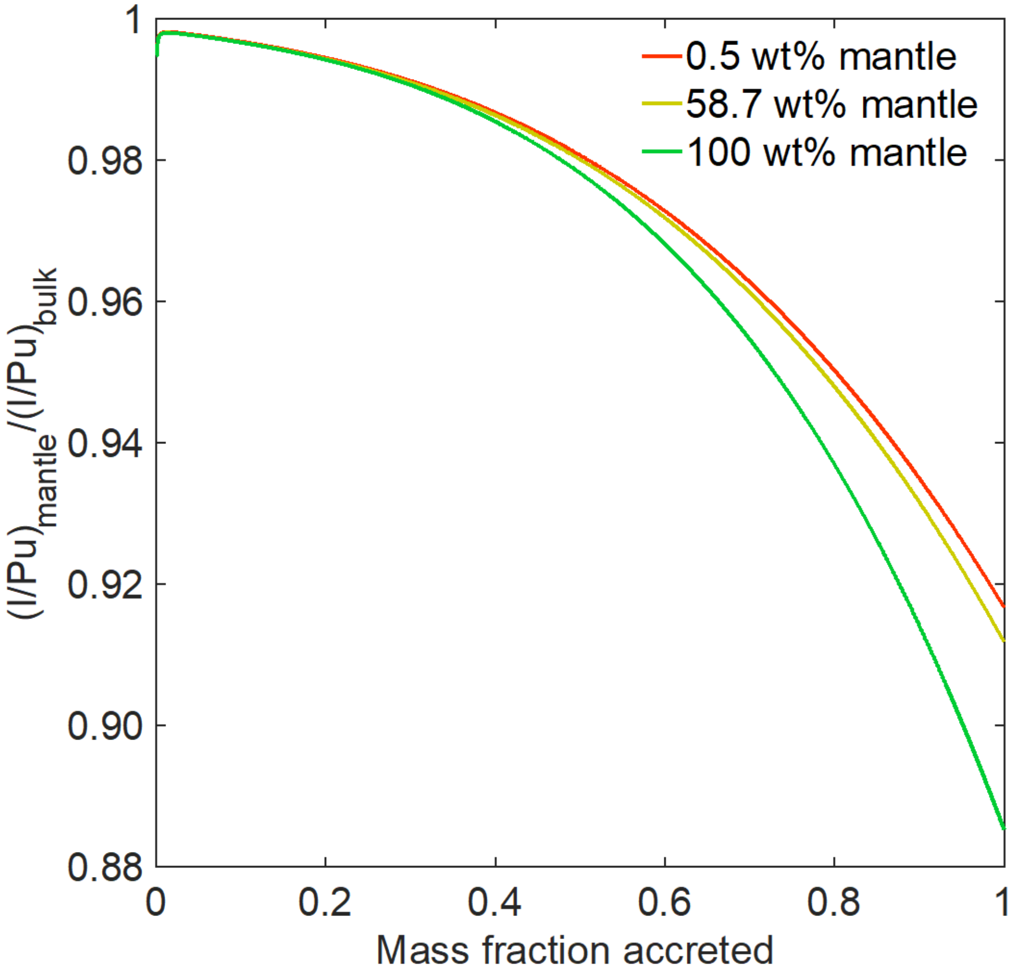

Figure S12. Variations of mantle I/Pu ratio as a function of the mass fraction accreted to the Earth with different portion of the proto-Mantle equilibrating with the metal liquid. The simulation is made under the fiducial model as described in Methods. The yellow ocher line shows the results when the metal liquid equilibrates with the magma ocean at the equilibrium pressure at the base of the magma ocean. The number of 58.7 wt% is determined by using equation (16) when P_{fin} is 60 GPa. The red line shows the results when the metal liquid equilibrates with 0.5 wt% mantle (under the same equilibrium pressure as the yellow line). The green line shows the results when the metal liquid equilibrates with the whole mantle (under the same equilibrium pressure as the yellow line). This figure shows that the fraction of the Earth's mantle involved in the magma ocean would not greatly impact the I/Pu ratios during differentiation.

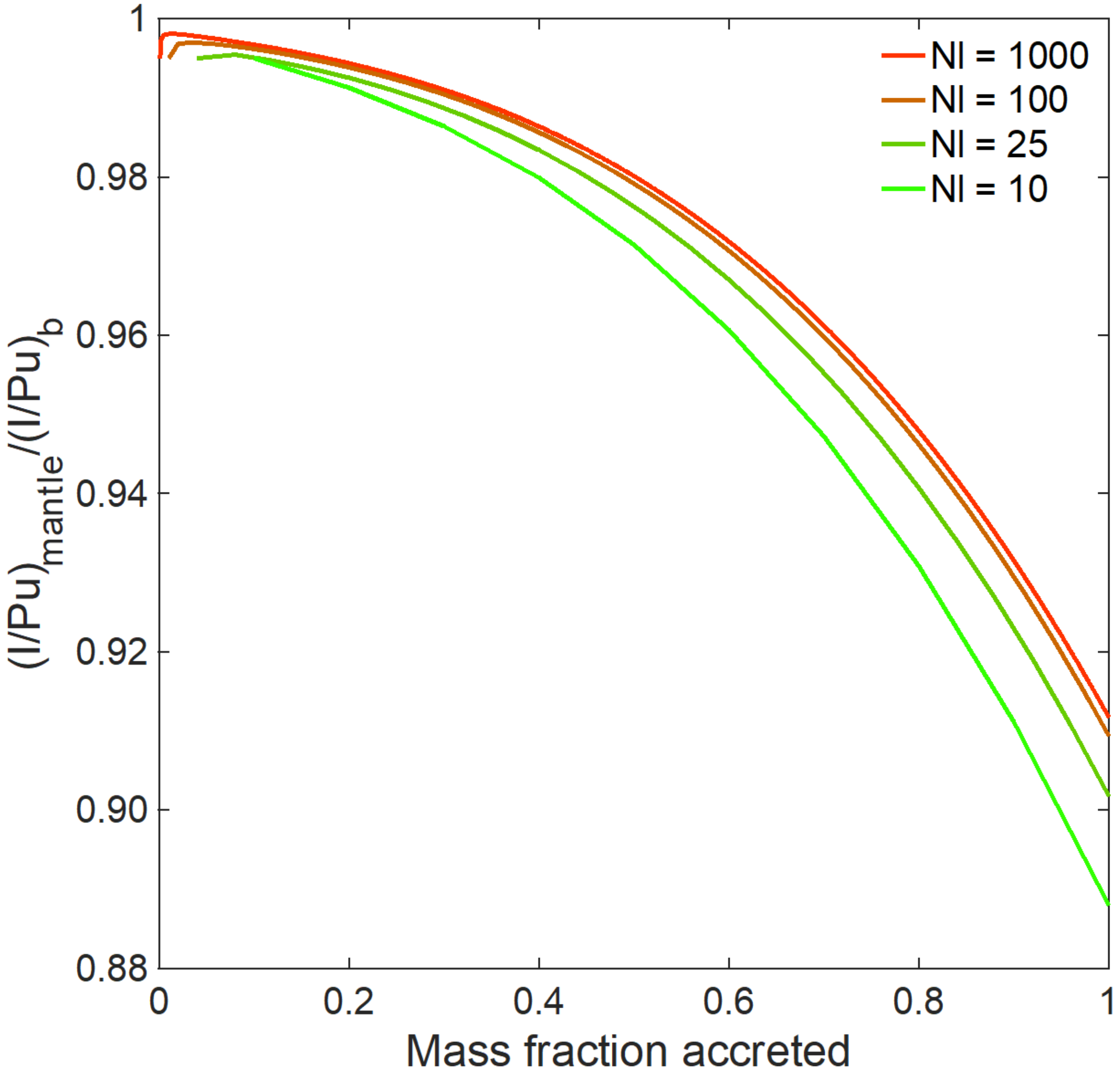

Figure S13. Variations of mantle I/Pu ratio as a function of the mass fraction accreted to the Earth with different number of accretion steps (Nl). The simulation is made under the fiducial model as described in Methods. Each increment of mass is 1/Nl for different model. Colors denote different number of steps. The number of step would not impact the I/Pu ratios during differentiation.

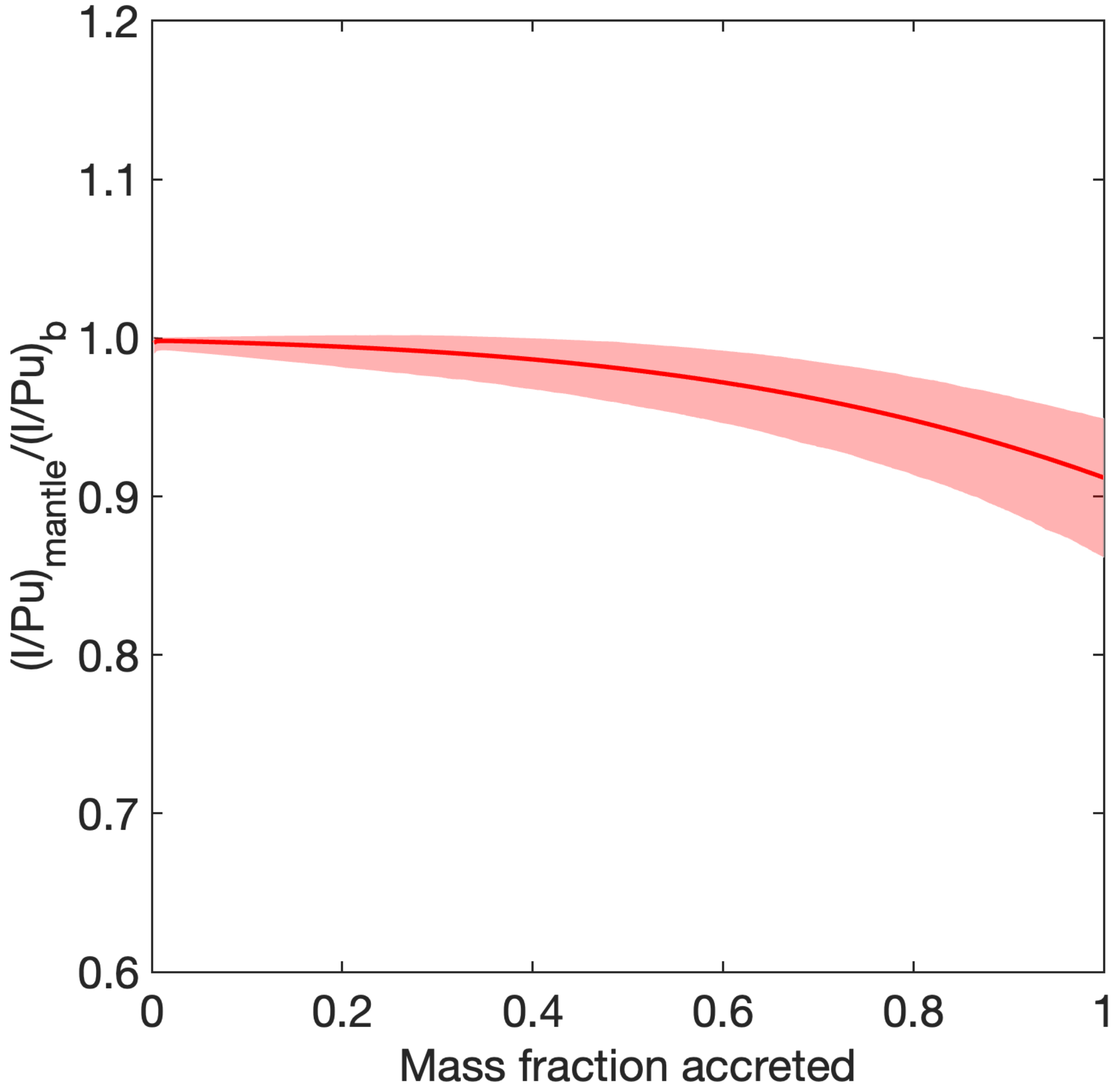

Figure S14. Variations of mantle I/Pu ratio as a function of the mass fraction accreted to the Earth with error envelopes. The simulation is made under the fiducial model as described in the Methods (same model run showed in Figure 2b). The red field corresponds to 1-sigma envelope of mantle I/Pu ratio variations using 1000 times Monte Carlo modelling. Uncertainties on partitioning coefficients are given in the Methods and covariance coefficient were as follows: $\sigma_{a_{Pu},b_{Pu}} = -1.85 * 10^3, \sigma_{a_I,b_I} = -6.70 * 10^3, \sigma_{a_I,c_I} = -23.2, \sigma_{b_I,c_I} = -9.61 * 10^3, \sigma_{a_I,\epsilon_{OI}} = 2.92, \sigma_{b_I,\epsilon_{OI}} = -9.07 * 10^3$ *and* $\sigma_{c_I,\epsilon_{OI}} = 35.7$.

Table S1 Results of first-principles molecular dynamics simulations on iodine partitioning.

			liquid iron (mol%)					silicate melt (mol%)				Oxygen fugacity
Runs	T(K)	P(GPa)	O	Si	Mg	Fe	I	SiO_2	MgO	FeO	I	$2\log(X_{FeO}/X_{Fe})$
I1	3641	69.6	10.42	4.84	0.79	79.70	4.24	34.37	54.71	8.67	2.25	-1.93
I2	3450	59.8	11.78	5.11	1.40	78.35	3.36	34.26	54.51	8.57	2.65	-1.92
I3	3247	50.3	3.46	8.78	0.39	85.57	1.80	37.26	56.03	3.25	3.46	-2.84

Table S2 Results of first-principles molecular dynamics simulations on plutonium partitioning.

Runs	T(K)	P(GPa)	liquid iron (mol%)					silicate melt (mol%)				Oxygen fugacity
			O	Si	Mg	Fe	Pu	SiO_2	MgO	FeO	$PuO_{3/2}$	$2\log(X_{FeO}/X_{Fe})$
Pu1	2804	24.6[1]	1.02	3.08	0.01	95.50	0.39	35.60	55.47	4.75	4.18	-2.61
Pu2	3450	53.6	9.39	9.13	0.72	79.96	0.80	32.70	55.64	7.68	3.99	-2.03
Pu3	4006	86.1	13.45	8.17	1.56	74.34	2.48	33.55	56.44	6.93	3.08	-2.06

Table S3 Xenon end-member compositions and meteoritic data used.

		^{132}Xe = 100								Reference
		^{124}Xe	^{126}Xe	^{128}Xe	^{129}Xe	^{130}Xe	^{131}Xe	^{134}Xe	^{136}Xe	
Endmember										
Spallogenic Xenon		71.69	120.5	183.1	192.8	118.1	454.2	5.301	0.3614	Ref. (*81*)
Solar Wind Xenon		0.4816	0.4222	8.412	104.1	16.49	82.63	36.98	29.99	Ref.(*82*)
Phase-Q Xenon		0.455	0.4057	8.22	104.2	16.19	81.85	37.8	31.64	Ref.(*83*)
Air Xenon		0.3537	0.33	7.136	98.32	15.14	78.9	38.79	32.93	Ref.(*84*)
Pu-fission Xenon		0	0	0	5.424	0	27.8	106.1	113	Ref.(*85*)
Uranium-fission Xenon		0	0	0	2.543	0	21.39	147.4	173.4	Ref.(*86*)
Meteorites										
Type	name									
CI	Ivuna	0.406 ±0.003	0.380 ±0.003	7.54 ± 0.05	125.6 ± 0.9	15.5 ± 0.1	79.9 ± 0.5	38.7 ± 0.2	32.8 ± 0.2	Ref. (*87*)
CO	KAINSAZ	0.4726 ± 0.0069	0.4151 ± 0.0065	8.316 ± 0.060	110.3 ± 0.4	16.27 ± 0.09	82.08 ± 0.26	38.01 ± 0.10	31.94 ± 0.07	Ref. (*88*)
CV	Bukhara	0.483 ± 0.003	0.425 ± 0.004	8.351 ±0.026	125.9 ± 0.2	16.35 ± 0.02	82.34 ± 0.11	37.83 ± 0.04	31.37 ± 0.13	Ref. (*89*)
CM	QUE 93005	0.4654 ± 0.0074	0.4037 ± 0.0054	8.107 ± 0.005	110.45 ±0.63	16.194 ±0.096	82.29 ± 0.50	38.81 ±0.26	32.70 ±0.24	Ref. (*90*)
H	GRV053690 -2	0.4812 ±0.0680	0.385 ±0.037	8.341 ±0.826	121.5 ±11.2	16.39 ±1.36	82.87 ±6.06	38.27 ±1.99	32.08 ±1.49	Ref. (*91*)
L	GRV052314 9-1	0.495 ±0.102	0.7591 ±0.1368	8.317 ±0.855	151.4 ±14.1	16.60 ±1.42	82.21 ±6.19	40.00 ±2.44	33.00 ±1.56	Ref. (*91*)
LL	St. Severin	0.511 ±0.014	0.529 ±0.018	8.007 ±0.073	137.0 ±1.6	15.74 ±0.08	80.92 ±0.29	38.60 ±0.16	32.52 ±0.12	Ref. (*92*)
EH	ALH 77295	0.4119 ±0.0040	0.3777 ±0.0026	7.829 ±0.019	137.34 ±0.14	15.644 ±0.027	80.25 ±0.10	38.838 ±0.054	33.023 ±0.051	Ref. (*93*)
EL	DAG 734	0.4322 ±0.0080	0.3747 ±0.0053	7.589 ±0.026	152.34 ±0.29	15.588 ±0.066	80.22 ±0.18	38.642 ±0.091	32.661 ±0.087	Ref. (*93*)
Angrites	Angra dos Reis (ρ > 3.2)	5.86 ±0.57	8.51 ±0.83	15.64 ±1.52	73.1 ± 0.7	13.9 ±1.1	79.1 ±7.3	62.80 ±6.03	62 ±6	Ref. (*94*)
Angrites	LEW 86010	7.1593 ±0.0623	11.3251 ±0.0896	18.519 ±0.129	58.89 ±0.27	14.303 ±0.102	76.406 ±0.349	74.515 ±0.300	77.567 ±0.286	Ref. (*95*)
Angrites	Sahara 99555 #3	0.638 ±0.018	0.695 ±0.012	7.02 ±0.12	90.8 ±0.9	14.08 ±0.19	75.0 ±0.8	45.1 ±0.5	40.1 ±0.5	Ref. (*96*)
Angrites	D'Orbigny #3	3.20 ±0.27	5.3 ±0.5	10.0 ±0.9	47 ±5	9.9 ±1.1	56 ±6	79 ±7	84 ±7	Ref. (*96*)
Angrites	NWA1296	0.479 ±0.013	0.514 ±0.018	7.69 ±0.05	96.4 ±0.3	14.93 ±0.23	77.2 ±0.4	44.3 ±0.3	39.1 ±0.2	Ref. (*97*)
Angrites	NWA2999	0.940 ±0.024	1.392 ±0.069	8.57 ±0.10	97.2 ±0.6	15.99 ±0.21	83.2 ±0.4	39.8 ±0.2	33.9 ±0.2	Ref. (*97*)
Angrites	NWA4590	7.937 ±0.211	12.679 ±0.705	20.12 ±0.33	49.3 ±0.7	15.20 ±0.28	71.0 ±0.7	77.4 ±1.1	80.4 ±0.8	Ref. (*97*)
Angrites	NWA4801	1.847 ±0.048	2.745 ±0.145	9.04 ±0.14	76.8 ±0.5	13.15 ±0.20	76.7 ±0.5	55.5 ±0.4	52.4 ±0.4	Ref. (*97*)
Angrites	NWA4931	3.798 ±0.146	5.783 ±0.372	14.48 ±0.27	88.5 ±0.9	16.99 ±0.29	94.3 ±0.7	49.3 ±0.5	46.3 ±0.4	Ref. (*97*)
HED	V1	1.64 ±0.41	3.18 ±0.36	13.91 ±0.41	97.24 ±0.36	20.87 ±0.30	91.79 ±0.15	44.63 ±1.11	42.06 ±1.63	Ref. (*98*)
HED	V2	0.91 ±0.18	1.49 ±0.13	9.09 ±0.05	98.65 ±1.02	17.99 ±0.13	85.36 ±0.84	40.59 ±0.16	34.36 ±0.28	Ref. (*98*)
HED	P1	1.91 ±0.04	2.84 ±0.02	10.16 ±0.08	94.86 ±0.38	16.42 ±0.06	83.68 ±0.32	43.15 ±0.13	38.32 ±0.19	Ref. (*98*)
HED	P2	10.4 ±0.02	2.52 ±0.03	9.67 ±0.12	93.12 ±0.13	16.24 ±0.04	81.81 ±0.21	43.98 ±0.19	38.86 ±0.18	Ref. (*98*)
HED	L1	6.96 ±0.03	10.68 ±0.31	21.1 ±0.3	86.1 ±0.5	20.70 ±0.52	95.44 ±2.08	55.25 ±0.45	50.48 ±0.73	Ref. (*98*)
HED	L2	8.08 ±0.09	9.83 ±0.12	20.18 ±0.92	104.8 ±1.1	18.15 ±0.69	87.39 ±0.51	50.70 ±1.13	44.96 ±1.31	Ref. (*98*)

Table S4 Solutions for radiogenic ^{129}Xe and plutonium-derived fissiogenic ^{136}Xe of different meteorites using Phase-Q as the initial composition. -1σ and +1σ give 68% confidence limits.

	(^{129}Xe*/^{132}Xe)			(^{136}Xe*$_{Pu}$/^{132}Xe)			(^{129}Xe*/^{132}Xe)/ (^{136}Xe*$_{Pu}$/^{132}Xe)	
Types	median	-1σ	+1σ	median	-1σ	+1σ	median	+1σ
Chondrites								
CI	0.2536	0.0092	0.0092	0.0031	0.0031	0.0036	80.60	38.76
CO	0.0629	0.0040	0.0040	0	-	-	-	-
CV	0.2167	0.0020	0.0020	0	-	-	-	-
CM	0.0707	0.0065	0.0065	0	-	-	-	-
H	0.1877	0.1128	0.1121	0.0000	0.0000	0.0048	-	63.10
L	0.5031	0.1423	0.1423	0.0000	0.0000	0.0105	-	61.66
LL	0.3559	0.0162	0.0162	0.0058	0.0031	0.0023	61.65	46.28
EH	0.3608	0.0016	0.0016	0.0127	0.0039	0.0009	28.31	26.50
EL	0.5172	0.0032	0.0032	0.0017	0.0017	0.0032	297.58	106.1
Achondrites								
Angra dos Reis	0.1083	0.0820	0.0812	0.5222	0.0514	0.0516	0.2074	0.3302
LEW 86010	0.0879	0.0035	0.0035	0.6888	0.0030	0.0029	0.1277	0.1322
Sahara 99555	0.0044	0.0044	0.0102	0.1103	0.0058	0.0062	0.0403	0.1258
D'Orbigny	0.0452	0.0452	0.0674	0.7320	0.0955	0.0577	0.0618	0.1425
NWA1296	0.0093	0.0058	0.0051	0.0930	0.0197	0.0107	0.0998	0.1384
NWA2999	0.0000	-	-	0.0239	0.0071	0.0072	-	-
NWA4590	0.0454	0.0097	0.0098	0.7571	0.0090	0.0090	0.0600	0.0721
NWA4801	0.0000	-	-	0.2867	0.0132	0.0068	-	-
NWA4931	0.0000	-	-	0.1317	0.0291	0.0295	-	-
V1	0.0000	-	-	0.0000	0.0000	0.0000	-	-
V2	0.0000	-	-	0.0000	0.0000	0.0059	-	-
P1	0.0035	0.0035	0.0049	0.0492	0.0093	0.0097	0.0710	0.1427
P2	0.0801	0.0019	0.0019	0.2327	0.0017	0.0017	0.3441	0.3495
L1	0.0518	0.0073	0.0071	0.3198	0.0060	0.0060	0.1619	0.1805
L2	0.3131	0.0132	0.0132	0.4016	0.0094	0.0094	0.7797	0.7940

-: indeterminate

Table S5. Solutions for radiogenic ^{129}Xe and plutonium-derived fissiogenic ^{136}Xe of different meteorites by using solar wind as initial composition. -1σ and +1σ give 68% confidence limits.

	(^{129}Xe*/^{132}Xe)			(^{136}Xe*$_{Pu}$/^{132}Xe)			(^{129}Xe*/^{132}Xe)/ (^{136}Xe*$_{Pu}$/^{132}Xe)	
Types	median	-1σ	+1σ	median	-1σ	+1σ	median	+1σ
Chondrites								
CI	0.2599	0.0092	0.0092	0.0000	0.0000	0.0097	-	27.60
CO	0.0757	0.0041	0.0042	0	-	-	-	-
CV	0.2260	0.0020	0.0021	0	-	-	-	-
CM	0.0852	0.0065	0.0065	0	-	-	-	-
H	0.1991	0.1125	0.1121	0.0000	0.0000	0.0175	-	17.78
L	0.5117	0.1417	0.1414	0.0000	0.0000	0.0177	-	36.96
LL	0.3653	0.0161	0.0161	0.0059	0.0059	0.0110	61.47	22.57
EH	0.3691	0.0015	0.0016	0.0000	0.0000	0.0022	-	164.8
EL	0.5234	0.0031	0.0031	0.0000	0.0000	0.0027	-	195.4
Achondrites								
Angra dos Reis	0.1085	0.0820	0.0812	0.5226	0.0515	0.0516	0.2076	0.3303
LEW 86010	0.0879	0.0035	0.0035	0.6888	0.0030	0.0029	0.1277	0.1322
Sahara 99555	0.0063	0.0063	0.0098	0.1140	0.0073	0.0078	0.0551	0.1324
D'Orbigny	0.0469	0.0469	0.0671	0.7337	0.0960	0.0579	0.0639	0.1439
NWA1296	0.0198	0.0074	0.0070	0.0818	0.0303	0.0267	0.2416	0.2467
NWA2999	0.0000	-	-	0.0251	0.0050	0.0062	-	-
NWA4590	0.0454	0.0097	0.0098	0.7571	0.0090	0.0090	0.0600	0.0721
NWA4801	0.0000	-	-	0.2874	0.0129	0.0067	-	-
NWA4931	0.0000	-	-	0.1321	0.0293	0.0299	-	-
V1	0.0000	-	-	0.0584	0.0255	0.0254	-	-
V2	0.0000	-	-	0.0365	0.0341	0.0127	-	-
P1	0.0036	0.0036	0.0049	0.0496	0.0095	0.0101	0.0716	0.1417
P2	0.0801	0.0019	0.0019	0.2327	0.0017	0.0017	0.3441	0.3495
L1	0.0510	0.0077	0.0073	0.3209	0.0065	0.0073	0.1588	0.1775
L2	0.3131	0.0132	0.0132	0.4016	0.0094	0.0094	0.7797	0.7940

-: indeterminate

REFERENCES AND NOTES

1. N. Dauphas, The isotopic nature of the Earth's accreting material through time. *Nature* **541**, 521–524 (2017).

2. D. C. Rubie, D. J. Frost, U. Mann, Y. Asahara, F. Nimmo, K. Tsuno, P. Kegler, A. Holzheid, H. Palme, Heterogeneous accretion, composition and core-mantle differentiation of the Earth. *Earth Planet. Sci. Lett.* **301**, 31–42 (2011).

3. C. Burkhardt, F. Spitzer, A. Morbidelli, G. Budde, J. H. Render, T. S. Kruijer, T. Kleine, Terrestrial planet formation from lost inner solar system material. *Sci. Adv.* **7**, eabj7601 (2021).

4. L. Piani, Y. Marrocchi, T. Rigaudier, L. G. Vacher, D. Thomassin, B. Marty, Earth's water may have been inherited from material similar to enstatite chondrite meteorites. *Science* **369**, 1110–1113 (2020).

5. A. N. Halliday, D. Porcelli, In search of lost planets – The paleocosmochemistry of the inner solar system. *Earth Planet. Sci. Lett.* **192**, 545–559 (2001).

6. C. Fitoussi, B. Bourdon, X. Wang, The building blocks of Earth and Mars: A close genetic link. *Earth Planet. Sci. Lett.* **434**, 151–160 (2016).

7. S. Mukhopadhyay, Early differentiation and volatile accretion recorded in deep-mantle neon and xenon. *Nature* **486**, 101–104 (2012).

8. M. K. Pető, S. Mukhopadhyay, K. A. Kelley, Heterogeneities from the first 100 million years recorded in deep mantle noble gases from the Northern Lau Back-arc Basin. *Earth Planet. Sci. Lett.* **369-370**, 13–23 (2013).

9. R. Parai, S. Mukhopadhyay, The evolution of MORB and plume mantle volatile budgets: Constraints from fission Xe isotopes in Southwest Indian Ridge basalts. *Geochem. Geophys. Geosyst.* **16**, 719–735 (2015).

10. A. Caracausi, G. Avice, P. G. Burnard, E. Füri, B. Marty, Chondritic xenon in the Earth's mantle. *Nature* **533**, 82–85 (2016).

11. C. R. M. Jackson, N. Bennett, Z. Du, E. Cottrell, Y. Fei, Early episodes of high-pressure core formation preserved in plume mantle. *Nature* **553**, 491–495 (2018).

12. J. M. Tucker, S. Mukhopadhyay, J.-G. Schilling, The heavy noble gas composition of the depleted MORB mantle (DMM) and its implications for the preservation of heterogeneities in the mantle. *Earth Planet. Sci. Lett.* **355-356**, 244–254 (2012).

13. S. Mukhopadhyay, R. Parai, Noble gases: A record of Earth's evolution and mantle dynamics. *Annu. Rev. Earth Planet. Sci.* **47**, 389–419 (2019).

14. M. W. Broadley, P. H. Barry, D. V. Bekaert, D. J. Byrne, A. Caracausi, C. J. Ballentine, B. Marty, Identification of chondritic krypton and xenon in Yellowstone gases and the timing of terrestrial volatile accretion. *Proc. Natl. Acad. Sci. U.S.A.* **117**, 13997–14004 (2020).

15. D. S. Musselwhite, M. J. Drake, Early outgassing of Mars: Implications from experimentally determined solubility of iodine in silicate magmas. *Icarus* **148**, 160–175 (2000).

16. R. M. G. Armytage, A. P. Jephcoat, M. A. Bouhifd, D. Porcelli, Metal–silicate partitioning of iodine at high pressures and temperatures: Implications for the Earth's core and ^{129*}Xe budgets. *Earth Planet. Sci. Lett.* **373**, 140–149 (2013).

17. M. G. Seitz, T. J. Gerding, M. J. Steindler, "Decontamination of metals containing plutonium and americium" (ANL-78-13, 5863065, Argonne National Lab, 1979).

18. Y. Zhang, Q.-Z. Yin, Carbon and other light element contents in the Earth's core based on first-principles molecular dynamics. *Proc. Natl. Acad. Sci. U.S.A.* **109**, 19579–19583 (2012).

19. Y. Zhang, G. Guo, Partitioning of Si and O between liquid iron and silicate melt: A two-phase ab initio molecular dynamics study. *Geophys. Res. Lett.* **36**, L18305 (2009).

20. W. Liu, Y. Zhang, Q.-Z. Yin, Y. Zhao, Z. Zhang, Magnesium partitioning between silicate melt and liquid iron using first-principles molecular dynamics: Implications for the early thermal history of the Earth's core. *Earth Planet. Sci. Lett.* **531**, 115934 (2020).

21. D. Andrault, N. Bolfan-Casanova, G. L. Nigro, M. A. Bouhifd, G. Garbarino, M. Mezouar, Solidus and liquidus profiles of chondritic mantle: Implication for melting of the Earth across its history. *Earth Planet. Sci. Lett.* **304**, 251–259 (2011).

22. J. Badro, J. Brodholt, H. Piet, J. Siebert, F. J. Ryerson, Core formation and core composition from coupled geochemical and geophysical constraints. *Proc. Natl. Acad. Sci. U.S.A.* **112**, 12310–12314 (2015).

23. R. A. Fischer, A. J. Campbell, F. J. Ciesla, Sensitivities of Earth's core and mantle compositions to accretion and differentiation processes. *Earth Planet. Sci. Lett.* **458**, 252–262 (2017).

24. I. Blanchard, J. Siebert, S. Borensztajn, J. Badro, The solubility of heat-producing elements in Earth's core. *Geochem. Perspect. Lett.*, **5**, 1–5 (2017).

25. B. A. Chidester, S. J. Lock, K. E. Swadba, Z. Rahman, K. Righter, A. J. Campbell, The lithophile element budget of Earth's core. *Geochem. Geophys. Geosyst.* **23**, 1–29 (2022).

26. G. Fiquet, A. L. Auzende, J. Siebert, A. Corgne, H. Bureau, H. Ozawa, G. Garbarino, Melting of peridotite to 140 gigapascals. *Science* **329**, 1516–1518 (2010).

27. K. Righter, C. D. K. Herd, A. Boujibar, Redox processes in early Earth accretion and in terrestrial bodies. *Elements* **16**, 161–166 (2020).

28. E. S. Steenstra, E. Kelderman, J. Berndt, S. Klemme, E. S. Bullock, W. van Westrenen, Highly reduced accretion of the Earth by large impactors? Evidence from elemental partitioning between sulfide liquids and silicate melts at highly reduced conditions. *Geochim. Cosmochim. Acta* **286**, 248–268 (2020).

29. K. Righter, M. J. Drake, E. Scott, *In Meteorites and the Early Solar System II*, D. S. Lauretta, H. Y. McSween, Eds. (Univ. Arizona Press, 2006).

30. A. Mundl, M. Touboul, M. G. Jackson, J. M. D. Day, M. Kurz, V. Lekic, R. T. Helz, R. J. Walker, Tungsten-182 heterogeneity in modern ocean island basalts. *Science* **356**, 66–69 (2017).

31. Z. Zhang, S. M. Dorfman, J. Labidi, S. Zhang, M. Li, M. Manga, L. Stixrude, W. F. McDonough, Q. Williams, Primordial metallic melt in the deep mantle. *Geophys. Res. Lett.* **43**, 3693–3699 (2016).

32. M. Schönbächler, R. W. Carlson, M. F. Horan, T. D. Mock, E. H. Hauri, Heterogeneous accretion and the moderately volatile element budget of Earth. *Science* **328**, 884–887 (2010).

33. U. Mann, D. J. Frost, D. C. Rubie, Evidence for high-pressure core-mantle differentiation from the metal-silicate partitioning of lithophile and weakly-siderophile elements. *Geochim. Cosmochim. Acta* **73**, 7360–7386 (2009).

34. Z. Wang, H. Becker, Ratios of S, Se and Te in the silicate Earth require a volatile-rich late veneer. *Nature* **499**, 328–331 (2013).

35. B. J. Peters, R. W. Carlson, J. M. D. Day, M. F. Horan, Hadean silicate differentiation preserved by anomalous $^{142}Nd/^{144}Nd$ ratios in the Réunion hotspot source. *Nature* **555**, 89–93 (2018).

36. E. Hyung, S. B. Jacobsen, The $^{142}Nd/^{144}Nd$ variations in mantle-derived rocks provide constraints on the stirring rate of the mantle from the Hadean to the present. *Proc. Natl. Acad. Sci. U.S.A.* **117**, 14738–14744 (2020).

37. P. L. Clay, R. Burgess, H. Busemann, L. Ruzié-Hamilton, B. Joachim, J. M. D. Day, C. J. Ballentine, Halogens in chondritic meteorites and terrestrial accretion. *Nature* **551**, 614–618 (2017).

38. D. V. Bekaert, M. W. Broadley, B. Marty, The origin and fate of volatile elements on Earth revisited in light of noble gas data obtained from comet 67P/Churyumov-Gerasimenko. *Sci. Rep.* **10**, 5796 (2020).

39. N. Dauphas, F. Robert, B. Marty, The late asteroidal and cometary bombardment of Earth as recorded in water deuterium to protium ratio. *Icarus* **148**, 508–512 (2000).

40. B. Marty, K. Altwegg, H. Balsiger, A. Bar-Nun, D. V. Bekaert, J.-J. Berthelier, A. Bieler, C. Briois, U. Calmonte, M. Combi, J. De Keyser, B. Fiethe, S. A. Fuselier, S. Gasc, T. I. Gombosi, K. C. Hansen, M. Hässig, A. Jäckel, E. Kopp, A. Korth, L. Le Roy, U. Mall, O. Mousis, T. Owen, H. Rème, M. Rubin, T. Sémon, C.-Y. Tzou, J. H. Waite, P. Wurz, Xenon isotopes in 67P/Churyumov-Gerasimenko show that comets contributed to Earth's atmosphere. *Science* **356**, 1069–1072 (2017).

41. F. L. H. Tissot, M. Collinet, O. Namur, T. L. Grove, The case for the angrite parent body as the archetypal first-generation planetesimal: Large, reduced and Mg-enriched. *Geochim. Cosmochim. Acta* **338**, 278–301 (2022).

42. A. Johansen, T. Ronnet, M. Bizzarro, M. Schiller, M. Lambrechts, Å. Nordlund, H. Lammer, A pebble accretion model for the formation of the terrestrial planets in the Solar System. *Sci. Adv.* **7**, eabc0444 (2021).

43. M. D. Ballmer, C. Houser, J. W. Hernlund, R. M. Wentzcovitch, K. Hirose, Persistence of strong silica-enriched domains in the Earth's lower mantle. *Nat. Geosci.* **10**, 236–240 (2017).

44. R. Arevalo, W. F. McDonough, M. Luong, The K/U ratio of the silicate Earth: Insights into mantle composition, structure and thermal evolution. *Earth Planet. Sci. Lett.* **278**, 361–369 (2009).

45. S. G. Nielsen, Potassium and uranium in the upper mantle controlled by Archean oceanic crust recycling. *Geology* **38**, 683–686 (2010).

46. G. Kresse, J. Furthmüller, Efficient iterative schemes for ab initio total-energy calculations using a plane-wave basis set. *Phys. Rev. B* **54**, 11169–11186 (1996).

47. P. E. Blöchl, Projector augmented-wave method. *Phys. Rev. B* **50**, 17953–17979 (1994).

48. G. Kresse, D. Joubert, From ultrasoft pseudopotentials to the projector augmented-wave method. *Phys. Rev. B* **59**, 1758–1775 (1999).

49. J. P. Perdew, K. Burke, M. Ernzerhof, Generalized gradient approximation made simple. *Phys. Rev. Lett.* **77**, 3865–3868 (1996).

50. P. Söderlind, A. Landa, B. Sadigh, Density-functional theory for plutonium. *Adv. Phys.* **68**, 1–47 (2019).

51. K. T. Moore, G. van der Laan, Nature of the 5*f* states in actinide metals. *Rev. Mod. Phys.* **81**, 235–298 (2009).

52. W. F. McDonough, Compositional model for the Earth's core, in *Treatise on Geochemistry* (Elsevier, 2014), pp. 559–577.

53. K. L. Clarkson, K. Mehlhorn, R. Seidel, Four results on randomized incremental constructions. *Comput. Geom.* **3**, 185–212 (1993).

54. O'Rourke, *Computational Geometry in C* (Cambridge University Press, ed. 2, 1998).

55. N. C. Hyatt, R. R. Schwarz, P. A. Bingham, M. C. Stennett, C. L. Corkhill, P. G. Heath, R. J. Hand, M. James, A. Pearson, S. Morgan, Thermal treatment of simulant plutonium contaminated materials from the Sellafield site by vitrification in a blast-furnace slag. *J. Nucl. Mater.* **444**, 186–199 (2014).

56. E. R. Vance, C. J. Ball, B. D. Begg, M. L. Carter, R. A. Day, G. J. Thorogood, Pu, U, and Hf incorporation in Gd silicate apatite. *J. Am. Ceram. Soc.* **86**, 1223–1225 (2003).

57. M. R. Cicconi, E. Pili, L. Grousset, P. Florian, J. C. Bouillard, D. Vantelon, D. R. Neuville, Iodine solubility and speciation in glasses. *Sci. Rep.* **9**, 7758 (2019).

58. R. A. Fischer, Y. Nakajima, A. J. Campbell, D. J. Frost, D. Harries, F. Langenhorst, N. Miyajima, K. Pollok, D. C. Rubie, High pressure metal-silicate partitioning of Ni, Co, V, Cr, Si, and O. *Geochim. Cosmochim. Acta* **167**, 177–194 (2015).

59. Z. Ma, Thermodynamic description for concentrated metallic solutions using interaction parameters. *Metall and Mater. Trans. B* **32**, 87–103 (2001).

60. J. Wade, B. J. Wood, Core formation and the oxidation state of the Earth. *Earth Planet. Sci. Lett.* **236**, 78–95 (2005).

61. J. Siebert, J. Badro, D. Antonangeli, F. J. Ryerson, Metal-silicate partitioning of Ni and Co in a deep magma ocean. *Earth Planet. Sci. Lett.* **321-322**, 189–197 (2012).

62. J. Siebert, J. Badro, D. Antonangeli, F. J. Ryerson, Terrestrial accretion under oxidizing conditions. *Science* **339**, 1194–1197 (2013).

63. W. F. McDonough, S. s. Sun, The composition of the Earth. *Chem. Geol.* **120**, 223–253 (1995).

64. K. Lodders, An oxygen isotope mixing model for the accretion and composition of rocky planets. *Space Sci. Rev.* **92**, 341–354 (2000).

65. K. Umemoto, K. Hirose, Chemical compositions of the outer core examined by first principles calculations. *Earth Planet. Sci. Lett.* **531**, 116009 (2020).

66. J. Badro, J. Siebert, F. Nimmo, An early geodynamo driven by exsolution of mantle components from Earth's core. *Nature* **536**, 326–328 (2016).

67. J. Badro, J. Aubert, K. Hirose, R. Nomura, I. Blanchard, S. Borensztajn, J. Siebert, Magnesium partitioning between Earth's mantle and core and its potential to drive an early exsolution geodynamo. *Geophys. Res. Lett.*, **45** (2018).

68. M. A. Bouhifd, A. P. Jephcoat, Convergence of Ni and Co metal–silicate partition coefficients in the deep magma-ocean and coupled silicon-oxygen solubility in iron melts at high pressures. *Earth Planet. Sci. Lett.* **307**, 341–348 (2011).

69. M. A. Bouhifd, D. Andrault, N. Bolfan-Casanova, T. Hammouda, J.-L. Devidal, Metal-silicate partitioning of Pb and U: Effects of metal composition and oxygen fugacity. *Geochim. Cosmochim. Acta* **114**, 13–28 (2013).

70. N. L. Chabot, D. S. Draper, C. B. Agee, Conditions of core formation in the Earth: Constraints from Nickel and Cobalt partitioning. *Geochim. Cosmochim. Acta* **69**, 2141–2151 (2005).

71. N. L. Chabot, C. B. Agee, Core formation in the Earth and Moon: New experimental constraints from V, Cr, and Mn. *Geochim. Cosmochim. Acta* **67**, 2077–2091 (2003).

72. A. Corgne, S. Keshav, B. J. Wood, W. F. McDonough, Y. Fei, Metal-silicate partitioning and constraints on core composition and oxygen fugacity during Earth accretion. *Geochim. Cosmochim. Acta* **72**, 574–589 (2008).

73. C. K. Geßmann, D. C. Rubie, The effect of temperature on the partitioning of nickel, cobalt, manganese, chromium, and vanadium at 9 GPa and constraints on formation of the Earth's core. *Geochim. Cosmochim. Acta* **62**, 867–882 (1998).

74. V. J. Hillgren, M. J. Drake, D. C. Rubie, High pressure and high temperature metal-silicate partitioning of siderophile elements: The importance of silicate liquid composition. *Geochim. Cosmochim. Acta* **60**, 2257–2263 (1996).

75. E. Ito, K. Morooka, O. Ujike, T. Katsura, Reactions between molten iron and silicate meltsat high pressure: Implications for the chemical. *J. Geophys. Res. Solid Earth* **100**, 5901–5910 (1995).

76. D. Jana, D. Walker, The impact of carbon on element distribution during core formation. *Geochim. Cosmochim. Acta* **61**, 2759–2763 (1997).

77. A. Ricolleau, Y. Fei, A. Corgne, J. Siebert, J. Badro, Oxygen and silicon contents of Earth's core from high pressure metal-silicate partitioning experiments. *Earth Planet. Sci. Lett.* **310**, 409–421 (2011).

78. T.-A. Suer, J. Siebert, L. Remusat, N. Menguy, G. Fiquet, A sulfur-poor terrestrial core inferred from metal-silicate partitioning experiments. *Earth Planet. Sci. Lett.* **469**, 84–97 (2017).

79. K. Tsuno, D. J. Frost, D. C. Rubie, Simultaneous partitioning of silicon and oxygen into the Earth's core during early Earth differentiation. *Geophys. Res. Lett.* **40**, 66–71 (2013).

80. Z. Du, C. Jackson, N. Bennett, P. Driscoll, J. Deng, K. K. M. Lee, E. Greenberg, V. B. Prakapenka, Y. Fei, Insufficient energy from MgO exsolution to power early geodynamo. *Geophys. Res. Lett.* **44**, 11,376–11,381.

81. C. M. Hohenberg, B. Hudson, B. M. Kennedy, F. A. Podosek, Xenon spallation systernatics in Angra dos Reis. *Geochim. Cosmochim. Acta* **45**, 1909–1915 (1981).

82. A. Meshik, O. Pravdivtseva, D. Burnett, Refined composition of Solar Wind xenon delivered by Genesis NASA mission: Comparison with xenon captured by extraterrestrial regolith soils. *Geochim. Cosmochim. Acta* **276**, 289–298 (2020).

83. H. Busemann, H. Baur, R. Wieler, Primordial noble gases in "phase Q" in carbonaceous and ordinary chondrites studied by closed-system stepped etching. *Meteorit. Planet. Sci.* **35**, 949–973 (2000).

84. J. R. Basford, J. C. Dragon, R. O. Pepin, M. R. Coscio Jr., V. R. Murthy, Krypton and xenon in lunar fines, in *Lunar and Planetary Science Conference Proceedings* (NASA Astrophysics Data System, 1973), vol. 4, p. 1915.

85. R. S. Lewis, Rare gases in separated whitlockite from the St. Severin chondrite: Xenon and krypton from fission of extinct 244Pu. *Geochim. Cosmochim. Acta* **39**, 417–432 (1975).

86. J. Macnamara, H. G. Thode, The isotopes of xenon and krypton in pitchblende and the spontaneous fission of U^{238}. *Phys. Rev.* **80**, 471–472 (1950).

87. M. E. I. Riebe, H. Busemann, R. Wieler, C. Maden, Closed system step etching of CI chondrite Ivuna reveals primordial noble gases in the HF-solubles. *Geochim. Cosmochim. Acta* **205**, 65–83 (2017).

88. L. Alaerts, R. S. Lewis, E. Anders, Isotopic anomalies of noble gases in meteorites and their origins—IV. C3 (Ornans) carbonaceous chondrites. *Geochim. Cosmochim. Acta* **43**, 1421–1432 (1979).

89. R. R. Mahajan, S. A. Ehgamberdiev, S. Naik, Noble gases and nitrogen in CV3 chondrite Bukhara. *Planet. Space Sci.* **207**, 105334 (2021).

90. D. Krietsch, H. Busemann, M. E. I. Riebe, A. J. King, C. M. O. Alexander, C. Maden, Noble gases in CM carbonaceous chondrites: Effect of parent body aqueous and thermal alteration and cosmic ray exposure ages. *Geochim. Cosmochim. Acta* **310**, 240–280 (2021).

91. Y. Wang, H. He, I. Leya, P. M. Ranjith, F. Su, P. C. Stephenson, C. Zhang, D. Zheng, The noble gases in five ordinary chondrites from Grove Mountains in Antarctica. *Planet. Space Sci.* **192**, 105045 (2020).

92. L. Alaerts, R. S. Lewis, E. Anders, Isotopic anomalies of noble gases in meteorites and their origins—III. LL-chondrites. *Geochim. Cosmochimica Acta* **43**, 1399–1415 (1979).

93. R. Okazaki, N. Takaoka, K. Nagao, T. Nakamura, Noble gases in enstatite chondrites released by stepped crushing and heating. *Meteorit. Planet. Sci.* **45**, 339–360 (2010).

94. G. J. Wasserburg, F. Tera, D. A. Papanastassiou, J. C. Huneke, Isotopic and chemical investigations on Angra dos Reis. *Earth Planet. Sci. Lett.* **35**, 294–316 (1977).

95. C. M. Hohenberg, T. J. Bernatowicz, F. A. Podosek, Comparative xenology of two angrites. *Earth Planet. Sci. Lett.* **102**, 167–177 (1991).

96. H. Busemann, S. Lorenzetti, O. Eugster, Noble gases in D'Orbigny, Sahara 99555 and D'Orbigny glass-evidence for early planetary processing on the angrite parent body. *Geochim. Cosmochim. Acta* **70**, 5403–5425 (2006).

97. D. Nakashima, K. Nagao, A. J. Irving, Noble gases in angrites Northwest Africa 1296, 2999/4931, 4590, and 4801: Evolution history inferred from noble gas signatures. *Meteorit. Planet. Sci.* **53**, 952–972 (2018).

98. R. R. Mahajan, A. B. Sarbadhikari, M. S. Sisodia, Noble gas, nitrogen composition and cosmic ray exposure history of two eucrites Vissannapeta, Piplia Kalan and one howardite Lohawat. *Planet. Space Sci.* **165**, 23–30 (2019).
